# Genomic Profiling of Childhood Tumor Patient-Derived Xenograft Models to Enable Rational Clinical Trial Design

**DOI:** 10.1016/j.celrep.2019.09.071

**Published:** 2019-11-05

**Authors:** Jo Lynne Rokita, Komal S. Rathi, Maria F. Cardenas, Kristen A. Upton, Joy Jayaseelan, Katherine L. Cross, Jacob Pfeil, Laura E. Egolf, Gregory P. Way, Alvin Farrel, Nathan M. Kendsersky, Khushbu Patel, Krutika S. Gaonkar, Apexa Modi, Esther R. Berko, Gonzalo Lopez, Zalman Vaksman, Chelsea Mayoh, Jonas Nance, Kristyn McCoy, Michelle Haber, Kathryn Evans, Hannah McCalmont, Katerina Bendak, Julia W. Böhm, Glenn M. Marshall, Vanessa Tyrrell, Karthik Kalletla, Frank K. Braun, Lin Qi, Yunchen Du, Huiyuan Zhang, Holly B. Lindsay, Sibo Zhao, Jack Shu, Patricia Baxter, Christopher Morton, Dias Kurmashev, Siyuan Zheng, Yidong Chen, Jay Bowen, Anthony C. Bryan, Kristen M. Leraas, Sara E. Coppens, HarshaVardhan Doddapaneni, Zeineen Momin, Wendong Zhang, Gregory I. Sacks, Lori S. Hart, Kateryna Krytska, Yael P. Mosse, Gregory J. Gatto, Yolanda Sanchez, Casey S. Greene, Sharon J. Diskin, Olena Morozova Vaske, David Haussler, Julie M. Gastier-Foster, Kolb E. Anders, Richard Gorlick, Xiao-Nan Li, C. Patrick Reynolds, Raushan T. Kurmasheva, Peter J. Houghton, Malcolm A. Smith, Richard B. Lock, Pichai Raman, David A. Wheeler, John M. Maris

**Affiliations:** 1 Division of Oncology, Children’s Hospital of Philadelphia, and Perelman School of Medicine at the University of Pennsylvania, Philadelphia, PA 19104-4318, USA; 2 Department of Bioinformatics and Health Informatics, Children’s Hospital of Philadelphia, Philadelphia, PA 19104, USA; 3 Center for Data-Driven Discovery in Biomedicine, Children’s Hospital of Philadelphia, Philadelphia, PA 19104, USA; 4 Human Genome Sequencing Center, Baylor College of Medicine, Houston, TX 77030, USA; 5 Guardian Forensic Sciences, Abington, PA 19001, USA; 6 UC Santa Cruz Genomics Institute, University of California, Santa Cruz, Santa Cruz, CA 95064, USA; 7 Cell and Molecular Biology Graduate Group, University of Pennsylvania, Philadelphia, PA 19104, USA; 8 Genomics and Computational Biology Graduate Group, University of Pennsylvania, Philadelphia, PA 19104, USA; 9 Department of Systems Pharmacology and Translational Therapeutics, University of Pennsylvania, Philadelphia, PA 19104, USA; 10 Children’s Cancer Institute, School of Women’s and Children’s Health, UNSW Sydney, Sydney, NSW, Australia; 11 Cancer Center, Texas Tech University Health Sciences Center School of Medicine, Lubbock, TX 79430, USA; 12 Sydney Children’s Hospital, Sydney, NSW, Australia; 13 Children’s Cancer Institute, Kensington, NSW, Australia; 14 Texas Children’s Cancer and Hematology Center, Department of Pediatrics, Baylor College of Medicine, Houston, TX 77030, USA; 15 Preclinical Neurooncology Research Program, Texas Children’s Cancer Research Center, Texas Children’s Hospital, Houston, TX 77030, USA; 16 Department of Pediatrics, Baylor College of Medicine, Houston, TX 77030, USA; 17 Department of Surgery, St. Jude Children’s Research Hospital, Memphis, TN 38105, USA; 18 Greehey Children’s Cancer Research Institute, University of Texas Health Science Center, San Antonio, TX 78229, USA; 19 The Research Institute at Nationwide Children’s Hospital, Columbus, OH 43205, USA; 20 Division of Pediatrics, The University of Texas MD Anderson Cancer Center, Houston, TX 77030, USA; 21 Department of Global Health Technologies, RTI International, Research Triangle Park, NC 27709, USA; 22 Department of Molecular and Systems Biology, Geisel School of Medicine at Dartmouth, Hanover, NH 03755, USA; 23 Norris Cotton Cancer Center, Lebanon, NH 03766, USA; 24 Childhood Cancer Data Lab, Alex’s Lemonade Stand Foundation, Philadelphia, PA 19102, USA; 25 Department of Molecular, Cell and Developmental Biology, University of California, Santa Cruz, Santa Cruz, CA 95064, USA; 26 Howard Hughes Medical Institute, University of California, Santa Cruz, Santa Cruz, CA 95064, USA; 27 The Ohio State University College of Medicine, Departments of Pathology and Pediatrics, Columbus, OH 43210, USA; 28 Department of Pediatrics, Sidney Kimmel Medical College at Thomas Jefferson University, Philadelphia, PA 19107, USA; 29 Nemours Center for Cancer and Blood Disorders, Nemours/Alfred I. duPont Hospital for Children, Wilmington, DE 19803, USA; 30 Division of Hematology, Oncology, Neuro-oncology and Stem Cell Transplant, Ann & Robert H. Lurie Children’s Hospital of Chicago, Chicago, IL 60611, USA; 31 Department of Pediatrics, Northwestern University Feinberg School of Medicine, Chicago, IL 60611, USA; 32 National Cancer Institute, NIH, Bethesda, MD 20814, USA; 33 Lead Contact

## Abstract

Accelerating cures for children with cancer remains an immediate challenge as a result of extensive oncogenic heterogeneity between and within histologies, distinct molecular mechanisms evolving between diagnosis and relapsed disease, and limited therapeutic options. To systematically prioritize and rationally test novel agents in preclinical murine models, researchers within the Pediatric Preclinical Testing Consortium are continuously developing patient-derived xenografts (PDXs)—many of which are refractory to current standard-of-care treatments—from high-risk childhood cancers. Here, we genomically characterize 261 PDX models from 37 unique pediatric cancers; demonstrate faithful recapitulation of histologies and subtypes; and refine our understanding of relapsed disease. In addition, we use expression signatures to classify tumors for *TP53* and *NF1* pathway inactivation. We anticipate that these data will serve as a resource for pediatric oncology drug development and will guide rational clinical trial design for children with cancer.

## In Brief

Rokita et. al provide an extensively annotated genomic dataset of somatic oncogenic regulation across 37 distinct pediatric malignancies. The 261 patient-derived xenograft models are available to the scientific community, and the genomic annotations will enable rational preclinical agent prioritization and acceleration of therapeutic targets for early-phase pediatric oncology clinical trials.

## Graphical Abstract

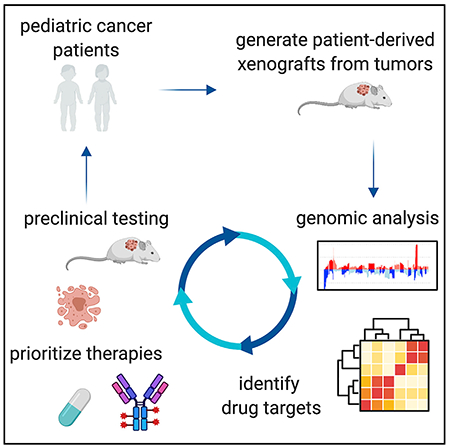

## INTRODUCTION

An estimated 15,780 children and adolescents (<20 years) are diagnosed with cancer in the United States each year, and these diverse entities are the leading cause of disease-related deaths in children (American Childhood Cancer Organization, 2014). Despite five-year survival rates for pediatric cancers now exceeding 80%, survivors frequently have lifelong side effects from cytotoxic therapy, and survival outcomes for children with certain types of tumors remain dismal. The relative rarity of pediatric cancers, molecular and mechanistic heterogeneity of subtypes within and across histologies, genetic and molecular distinction from adult malignancies, tumor evolution in the face of cytotoxic standard therapies, and lack of targeted therapeutic agents all pose major challenges to improving outcomes for children with cancer. Indeed, there are very few drugs with specific labeled indications for pediatric malignancies, and most standard therapies are largely empiric.

Preclinical testing of new therapeutic anti-cancer agents is essential in the field of pediatric oncology due to the relative rarity of the condition and the need to prioritize agents for early-phase clinical trials. Over the past 15 years, the Pediatric Preclinical Testing Consortium (PPTC), previously known as the Pediatric Preclinical Testing Program (Houghton et al., 2002, 2007), has developed over 370 patient-derived xenograft (PDX) models from high-risk childhood cancers. In collaboration with pharmaceutical and academic partners, the PPTC systematically screens novel therapeutic agents for anti-tumor efficacy in order to help prioritize those that will move to the clinic. Previous studies have characterized subsets of pediatric xenograft models, often with limited numbers of specific histologies and/or genomic assays (Brabetz et al., 2018; El-Hoss et al., 2016; Stewart et al., 2017; Townsend et al., 2016; Whiteford et al., 2007). Here, we present a comprehensive genomic characterization of 261 models from 29 unique pediatric cancer malignancies.

## RESULTS

### Genomic Analysis Workflow and Histological Summary of Pediatric PDX Tumors

**Figure 1.**
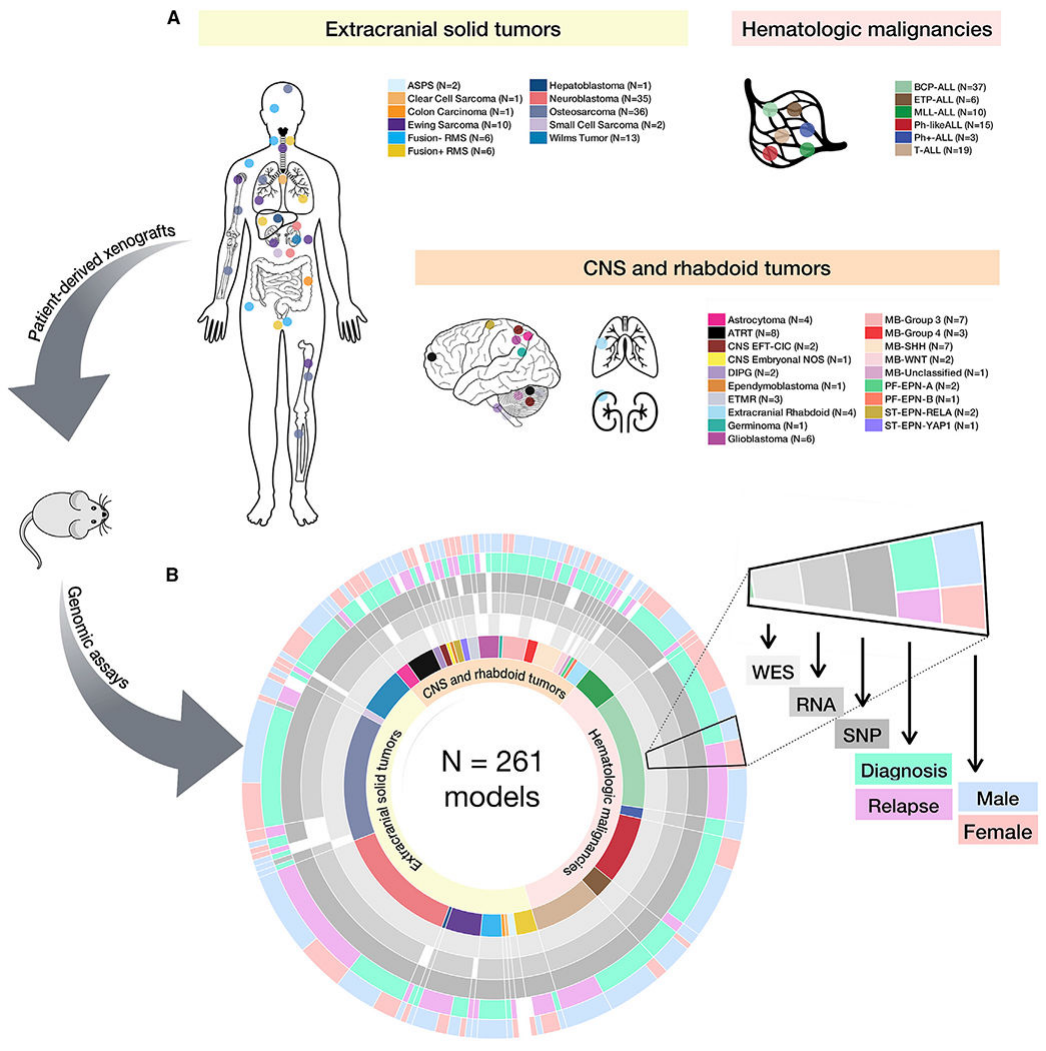
Study and Sample Overview (A and B) Diversity of the 261 childhood tumors collected (A) and demographics and genomic assays performed by histology (B). Assays performed were whole-exome sequencing (n = 240), whole transcriptome (n = 244), and SNP array copy number analysis (n = 252). Each genomic assay was performed once per biological tumor sample. See Figure S1 for analysis pipelines, Table S1 for model metadata, and Table S2 for STR profiles.

Figure 1 depicts the overall workflow of our study, including model histologies, site of tumor specimen, phase of therapy, and molecular assays performed. The PDX generation methods are described in the STAR Methods. We performed whole-exome sequencing (WES) on 240 childhood cancer PDX models, whole-transcriptome sequencing (RNA sequencing [RNA-seq]) on 244 models, and SNP microarrays on 252 models (Figures 1 and S1; Table S1), and we performed short tandem repeat (STR) profiling on all 261 models (Table S2). Of the 261 models profiled, 82 had available references that are also included in Table S2.

Figure S1 describes the analysis workflow (see STAR Methods for details). Of the 240 models on which WES was performed, 69 models were previously sequenced through efforts of the PPTP (dbGAP: phs000469.v17.p7), and we harmonized these data. For WES (Figure S1C) and RNA-seq (Figure S1D), we performed competitive mapping to a hybrid human-mouse reference (hg19-mm10) and used human-specific BAM files as input for downstream analyses. We validated this biochemically with qPCR by calculating the ratio of human:mouse DNA in a subset of 35 PDX tumors. We found a significant correlation between the percent of human reads following WES hybrid mapping and the percent of human DNA in the tumor extract (Figure S1B; Pearson correlation R = 0.943, F = 272.5, df = 34, p value < 2.2e–16). A mutation annotation format (MAF) file of common germline variation was created if a variant was present in more than five normal samples from The Cancer Genome Atlas (TCGA) patients (n = 809). The remaining variants, comprising both somatic and rare germline alterations, were collated into the “somatic” MAF file. Artifactual sequencing variants were removed as described in the STAR Methods. Common germline SNP distributions (allele frequency > 0.005 in any one of the three databases: Exome Aggregation Consortium, 1,000 genomes, or the NHBLI Exome Sequencing Project) were plotted for each model and visually inspected for a negatively skewed distribution to assess DNA cross-contamination in WES data. To identify potential misidentification, RNA variant calling was performed, and variant allele frequencies were correlated between WES and RNA. Models whose variants did not correlate were deemed misidentified and removed (STAR Methods). Within this cohort, five pairs of models were derived from tissue at the phase of therapy (Table S1). Thus, as additional quality control (QC), we correlated somatic mutation allele frequencies between each pair and found a high concordance of mutation frequencies (data on Figshare; STAR Methods), confirming the biological reproducibility of creating PDX models within a center. Mutation variation is summarized per model in Table S3.

SNP arrays were processed for segmentation, focal copy number, and ethnicity inference (STAR Methods; Figures S1A and S2). As reported ethnicities were only available for a small proportion of the models, we used SNP array genotypes to infer approximate ethnicities using HapMap genotype frequencies. We assigned models to African, East Asian, European, and South Asian/Hispanic ethnicities (Figure S2; Table S1). Overall, 71% of models are of predicted European descent, 11.5% South Asian/Hispanic, 9.1% African, 5.5% mixed or unknown ethnicity, and 2.4% East Asian.

Following rigorous assessment for contamination, misidentification, and sample mislabeling, 26 full models were excluded, and 3 RNA samples were excluded. The remaining 261 models used herein were shown to be free of detectable levels of DNA contamination (STAR Methods).

### PDX Models Recapitulate the Mutation and Copy-Number Landscape of Childhood Cancers

**Figure 2.**
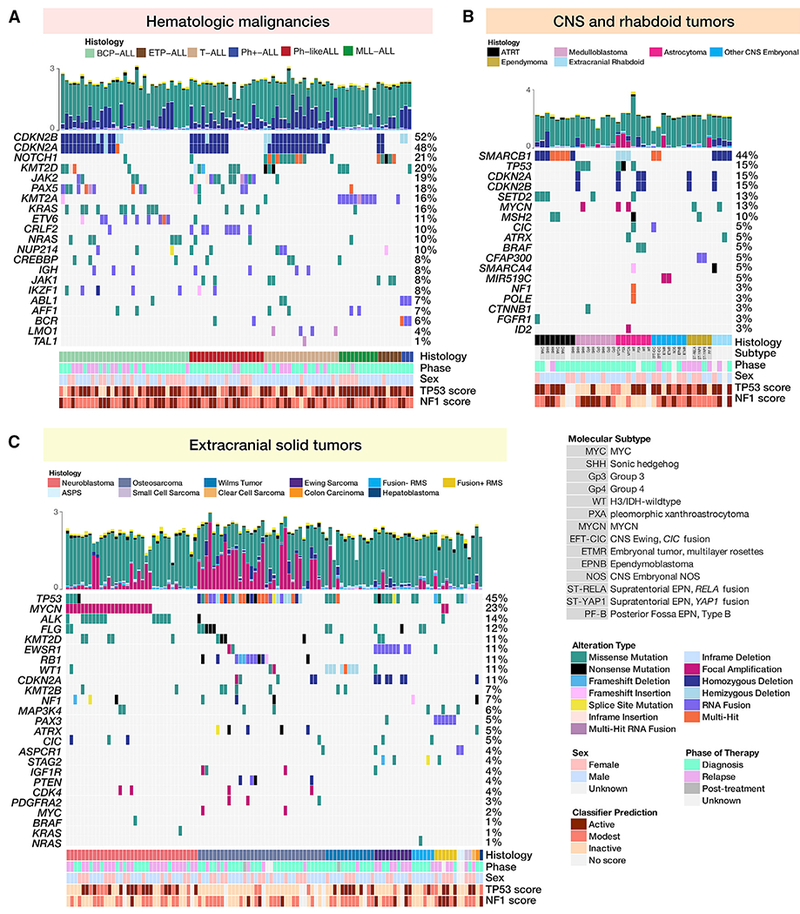
PDX Models Recapitulate the Mutational Landscape of Childhood Cancers (A–C) Oncoprints of somatic alterations (homozygous deletions, amplifications, SNVs, and fusions) in hallmark driver genes for PDX models for which exome sequencing was performed (n = 240, top 20 genes per histology shown). Oncoprints are grouped by acute lymphoblastic leukemias (A), CNS and rhabdoid tumors (B) and extracranial solid tumors (C). (A) From left to right are B cell precursor ALLs (n = 33), T cell ALLs (n = 25), Philadelphia chromosome positive (Ph+) ALLs (n = 3), mixed lineage leukemias (MLL, n = 10), early T cell precursor (ETP) ALLs (n = 6), and Philadelphia chromosome-like (Ph-like) ALLs (n = 19). (B) From left to right are atypical teratoid rhabdoid tumors (ATRTs; n = 8), medulloblastomas (MBs; n = 8), astrocytomas (n = 7), non-MB/non-ATRT CNS embryonal tumors (n = 7), ependymomas (n = 5), and extracranial rhabdoid tumors (n = 4). (C) From left to right are neuroblastomas (n = 35), osteosarcomas (n = 34), Wilms tumors (n = 13), Ewing sarcomas (n = 10), fusion negative rhabdomyosarcomas (n = 6), fusion positive rhabdomyosarcomas (n = 6), and rare solid tumors (n = 7). Clinical annotations for all models include histology, patient phase of therapy from which PDX was derived, and sex. CNS tumors were also annotated with molecular subtype. Hemizygous deletions in *TP53* are annotated for osteosarcoma models, in *CDKN2A* for leukemia models, and in *WT1* for Wilms tumor models. Focal homozygous deletions correspond to loss of expression (FPKM < 1) in models for which RNA was available. For fusions, only the 5′ partner is shown. Total mutations (log 10) per model are plotted above each oncoprint and colored by mutation type. Each genomic assay was performed once per biological tumor sample.

We highlight hallmark alterations in key pediatric tumor driver genes (Behjati et al., 2017; Eleveld et al., 2015; Gröbner et al., 2018; Liu et al., 2017; Ma et al., 2018; Pugh et al., 2013; Shern et al., 2014; Zhang et al., 2012) in Figure 2 and demonstrate faithful disease recapitulation across PDX models.

#### Acute Lymphoblastic Leukemias

Figure 2A depicts oncoprints for 90 acute lymphoblastic leukemia models.

#### BCP ALLs

A total of 45%–48% of B cell precursor acute lymphoblastic leukemia (BCP-ALL) PDX models contain canonical focal deletions of the tumor suppressors on chromosome 9p, *CDKN2A* or *CDKN2B* (Figures 2A and S4B), the majority of which are homozygous. The BCP-ALL models were enriched for alterations in the RAS pathway (*KRAS* mutated in 30%, *NRAS* mutated in 18%) and the JAK-STAT pathway (*JAK2/3* altered in 15%), and 15% have altered *KMT2D*. These pathways, along with *PI3K/AKT, TNFα*, and *TP53* signaling, were all significantly enriched in gene expression data (Figure 5B). Finally, we detected fusion transcripts in 78% of BCP-ALL models (25/33), many of which contain *ETV6* (27%; 88% of these partner with *RUNX1), PAX5* (18%), and *CRLF2* (6%) (Table S5).

#### ETP and T-ALLs

Early T cell precursor-ALL (ETP-ALL) and T cell-ALL (T-ALL) models are predominantly characterized by *CDKN2A/B* focal deletions (72%–76%; Figure S4B) and/or a *NOTCH1* mutation (68%). Genes within the JAK-STAT pathway are also frequently altered with concurrent pathway enrichment (Figure 5B). *JAK1 or JAK2* lesions were observed in 24% of the models, and 4% of the models contain lesions in *STAT5B*. We detected oncogenic fusion transcripts in nearly half (48%) of these models, many partnering with the following genes: *TRBC2* (16%), *TRBC1* (12%), *ABL1* (8%), *IGH* (8%), *LMAN2* (4%), *LMO1* (4%), *LMO2* (4%), and *ETV6* (4%).

#### Ph-like and Ph+ ALLs

We confirmed the presence of a *BCR-ABL1* fusion in all three Ph+-ALL models (ALL-04, ALL-55, and ALL-56). Eight Ph-like ALL models (42%; 10/19) contain a canonical *CRLF2* fusion; seven partner with *P2RY8* and one with *IGHM*. Additional frequently rearranged genes include *JAK2* (55%; 12/22) and *PAX5* (23%; 5/22). In both Ph+ and Ph-like ALL models, focal deletions of CDKN2A/B (45%, 10/22; Figure S4B) are predominant. Frequently altered pathways include Ras and JAK-STAT (Figures 2A and 5B).

#### MLL-ALLs

All mixed lineage leukemia-ALL (MLL-ALL) models contain a canonical *KMT2A* fusion and have relatively silent genomes with minimal copy number alterations (Figure S4B). The majority of these models were derived from children <1 year of age (Table S1).

### Molecular Subtyping and Genomic Landscape of CNS Tumors

Models derived from CNS and extracranial rhabdoid tumors were further molecularly classified using pathology reports or genomic features from WES, RNA, and SNP arrays (Figure 1; Table S1). Atypical teratoid rhabdoid tumor (ATRT) models represented both Sonic hedgehog (SHH; n = 3) and MYC (n = 3) subgroups, with two models unclassified. To classify medulloblastoma models, we developed and applied a classifier for RNA-seq data (STAR Methods). The 20 medulloblastoma models in this cohort span all broad subtypes: SHH (n = 7), WNT (n = 2), group 3 (n = 7), and group 4 (n = 3), and one model without RNA-seq remained unclassified. Other CNS embryonal tumors were classified into embryonal tumor with multi-layer rosettes (ETMR; n = 3), CNS Ewing with *CIC* alteration (CNS EFT-CIC; n = 2), ependymoblastoma (n = 1), or CNS embryonal not otherwise specified (CNS embryonal not otherwise specified [NOS]; n = 1). Astrocytoma models comprised pleomorphic xanthroastrocytomas (PXA; n = 2), MYCN subtype (n = 2), glioblastomas (IDH-wild-type; n = 5), histone H3-wild-type diffuse intrinsic pontine glioma (DIPG; n = 2), and a histone H3-wild-type astrocytic tumor (n = 1). Ependymal tumors were classified into supratentorial *RELA* positive (ST-EPN-RELA; n = 2), supratentorial *YAP1* positive (ST-EPN-YAP; n = 2), posterior fossa type A (PF-EPN-A; n = 1), or posterior fossa type B (PF-EPN-B; n = 1), and one remained unclassified.

All ATRT and extracranial rhabdoid models harbor inactivating alterations (focal deletion, frameshift deletion, or nonsense mutation) in the hallmark tumor suppressor, *SMARCB1*, and/or *SMARCA4*. Hedgehog, *TNFα*, and p53 signaling were enriched in these models (Figure 5B). Interestingly, three astrocytic tumors harbored *SMARCB1* hemizygous deletions, which have not been reported but are present in multiple pediatric high-grade glioma cohorts (Mackay et al., 2017: 6.7%, n = 834; Ijaz et al., 2019: 7.5%, n = 93) and may warrant further investigation. One astrocytic model, IC-1621GBM, was generated from a patient with DNA mismatch repair deficiency syndrome and showed 124 somatic mutations per medulloblastoma (MB) (Table S3). We confirmed multiple mutations in mismatch repair genes *PMS1, MSH2, MSH5*, and *POLE* (non-exonuclease domain mutation). The likely oncogenic drivers are the nonsense mutations in *PMS1* (Q316*) and *MSH2* (G721*), which disrupt the DNA mismatch repair protein domain and the MutS domain, respectively (Figure S3C). NCH-MN-1 was derived from a patient diagnosed with an anaplastic rhabdoid meningioma with the clinical suspicion of an ATRT; however, this model had no evidence of an inactivating *SMARCB1* alteration. Rather, it harbors a *BRAF* V600E mutation and focal *CDKN2A/B* deletion, classifying this model as a high-grade glioma, herein denoted as an astrocytoma. Not surprisingly, astrocytoma and glioblastoma models had similar pathway enrichment: estrogen response, hedgehog signaling, protein secretion, *TNFα*, and p53 pathway (Figure 5B).

IC-2664PNET was derived from a patient diagnosed with a primitive neuroectodermal tumor (PNET) but was further molecularly classified as a MYCN-subtype high-grade glioma. IC-2664PNET has a focal amplification of *MYCN* and a hemizygous *SMARCB1* deletion, but it retains mRNA expression of *SMARCB1*. ICb-S1129MB, ICb-1343ENB, and IBs-2373PNET were classified as ETMRs due to an amplification of C19MC, an overexpression of *LIN28A*, and/or *TTYH1* fusions. ICb-9850PNET and IC-22909PNET-rIII, a diagnosis-relapse pair, were genetically classified as CNS EFT-CIC, as the diagnostic tumor contains a *CIC-DUX4* fusion. The two DIPG models were profiled with RNA-seq and SNP arrays and thus are not shown in the oncoprint. We confirmed both IBs-P1215DIPG and IBs-W0128DIPG have high expressions of *H3F3A* and *H3F3B* (FPKM > 50), genes encoding the histone H3.3 variant, and lack expressions of *HIST1H3B* or *HIST1H3C*, genes encoding the histone H3.1 variant. While we did not detect H3.1 or H3.3 histone mutations in these models, RNA variant calling revealed IBs-W0128DIPG contained predicted damaging (PolyPhen) missense mutations in *NRAS* (p.G13R, 0.41), *CIC* (p.C102Y, 0.44), and *KMT2C* (p.C988F, 0.45). We did not detect any hallmark damaging mutations in IBs-P1215DIPG.

### Extracranial Solid Tumors

#### Neuroblastomas

Amplification of the *MYCN* oncogene was the most frequent alteration observed across all models (66%) and, as expected, was largely mutually exclusive of 11q deletion. Gene set enrichment analysis (GSEA) confirmed the enrichment of MYC targets in these models (Figure 5B). A majority (77%) of models had 1p deletion and 17q gain (97%; collapsed profiles are shown in Figure S4A). Consistent with previous reports (Pugh et al., 2013), we find *ALK* to be the most frequently mutated gene (37% of all models contain hotspot mutations) with additional, less frequent alterations in hallmark genes such as *TP53* (11%), *PTPN11* (9%), *NF1* (9%), *BRAF* (3%), *CIC* (3%), and *KRAS* (3%). The nonsense and frameshift deletions in *NF1* correspond with ablated expressions in COG-N-590x and NB-1771, respectively, but NB-1643 retains expression.

#### Osteosarcomas

The hallmark of osteosarcomas is *TP53* inactivation, and using a classifier trained on RNA expression data from TCGA, we found all osteosarcoma models with available RNA-seq data (n = 32) were predicted to have non-functional *TP53* (described below). Thus, as expected, *TP53* was the most commonly altered gene (82%) in osteosarcoma PDX models (Figure 2C), which also demonstrate global copy number changes, consistent with the high prevalence of complex genomic rearrangements found in this tumor type (Figure S4).

#### Ewing Sarcomas

The canonical *EWSR1-FLI1* fusion was found in all Ewing sarcoma models profiled with RNA-seq (NCH-EWS-1 was not profiled), and CHLA-258 contained an additional *FLI1* fusion partner: *RP11-9L18.2* (Table S5; Figure 2C). *TP53* mutations are present in seven cases (70%), with six showing allele frequencies at or near 1.0 due to copy-neutral loss of heterozygosity (cnLOH, ES-6, EW-8, and SK-NEP-1) or loss of heterozygosity (LOH) from a chromosomal arm deletion (EW-5, ES-8, and TC-71). Homozygous *CDKN2A/B* loss (60%) was mutually exclusive to *STAG2* mutations (20%), as expected (Tirode et al., 2014). We observe canonical (Tirode et al., 2014) broad gain of whole chromosomes 8 and 12, as well as focal 1q gain and 16q loss, in Ewing sarcomas (Figure S4A).

#### Wilms Tumors

The mutational and copy number landscapes of Wilms tumor (n = 13) PDX models are depicted in Figures 2C and S4A. The *WT1* gene located at 11p13 was mutated in one PDX model (NCH–WT–6–S13–1506), but we observed hemizygous deletions of *WT1* in 61% of Wilms models, many of which had LOHs of the entire 11p13 region. The 11p15.5 region, which contains imprint control regions (ICRs) 1 and 2, often undergoes loss of imprinting (LOI) either due to maternal DNA methylation or maternal LOH/paternal uniparental disomy (pUPD) in a Wilms tumor. The 11p15.5 region harbored LOHs in 69% (9/13) of Wilms tumors, consistent with previous reports (Scott et al., 2012). Two models (15%) harbored hemizygous deletions of *AMER1* (formerly known as *WTX* and/or *FAM123B)*. KT-9 is the only model annotated as coming from a patient with bilateral disease, and although it does not harbor a *WT1* mutation, interestingly, it has two hits in *TP53*: a *TP53-FXR2* fusion and a partial homozygous deletion. The Wilms models (15%; KT-6 and NCH-WT-6-S13-1506) with *CTNNB1* mutations were mutually exclusive to *WTX* alterations, consistent with previous reports (Scott et al., 2012). Gains of the 1q arm, 1p LOH, and 16q LOH–adverse prognostic biomarkers for Wilms tumors (Pan et al., 2017; Segers et al., 2013; Spreafico et al., 2013)–were observed in 31% (4/13), 8% (1/13), and 23% (3/13) of models, respectively (Figure S4).

#### Rhabdomyosarcomas

All Fusion+ rhabdomyosarcoma (RMS) models harbored a hallmark *PAX3-FOXO1* fusion (Figure 2C; Table S1), and the median patient age of Fusion+ RMS patients (16 years) was higher than that of Fusion− RMS patients (5 years) (Table S3). As expected, we also observed focal amplifications of *MYCN* and *CDK4*. Interestingly, the amplification of *CDK4* was not retained in Rh-30R (relapse tumor paired with Rh-30; SNPs and STRs confirm identity). Ras pathway mutations (*NRAS, HRAS, KRAS*, and *NF1*) are typically observed in one-third of Fusion− RMS cases and here, Ras mutations were observed in 3/6 models (Rh-12 with *NF1 T2335fs*, NCH–ERMS–1–NCH–RMS–1 with *NRAS Q61K* mutation, and Rh-36 with *HRAS* Q61K). Of note, all models except for IRS-68 overexpress the common rhabdomyosarcoma biomarker, *MYOD1*.

#### Rare Histologies

Seven PDX models were derived from rare tumor types and are depicted in Figure 2C. Three models (43%) contained alterations in *TP53*; of note, an in-frame hemizygous deletion of *TP53* evolved at the relapse in NCH-CA-2 (not present in diagnostic model, NCH-CA-1). The canonical *ASPSCR1-TFE3* fusion was detected in both alveolar soft part sarcoma (ASPS) models. NCH-CA-1 and NCH-CA-2 harbored deleterious *SMARCA4* mutations, and NCH-CA-3 harbored a deleterious *NF1* nonsense mutation; each had a concurrent loss of mRNA expression and, as such, these may be potential drivers of oncogenesis in these tumors. NCH-HEP1 contained a likely oncogenic WNT pathway mutation (*CTNNB1* p.D32G).

### Breakpoint Density

We calculated the total number of breakpoints per sample and breakpoint density within chromosomes, the latter as a surrogate measure of putative chromothripsis events (STAR Methods). Consistent with pediatric cancer genomics literature, we observed very few breakpoints per sample in hematologic malignancies, compared to those in solid tumors (median = 3 breakpoints per sample in CNS embryonal NOS to median = 154.5 breakpoints per sample in osteosarcoma; Figure S4C; Table S3). We found 25% (64/252) of models profiled have a high breakpoint density (HBD) across one or more chromosomes (Figure S4D; Table S3), consistent with a recent pan-cancer chromothripsis report (Cortes-Ciriano et al., 2018). Specifically, 97% (33/34) of osteosarcomas had HBDs; 30% (10/33) of these contained HBDs on four or fewer chromosomes indicative of localized chromothripsis events, while the remaining 70% (23/33) contained HBDs on five or more chromosomes, supporting the globally rearranged genomes prevalent in this tumor type (Lorenz et al., 2016). In neuroblastoma samples, 17% of models contained HBDs on chromosomes 2, 5, 16, 17, and 19. Chromothripsis events on chromosomes 2, 5, and 17 in neuroblastoma tumors have been previously reported to be associated with *MYCN* amplification, *TERT* rearrangements, and 17q gain, respectively (Molenaar et al., 2012; Boeva et al., 2013). Recurrent loci with HBDs in medulloblastoma were chromosomes 2, 8, 14, and 17, consistent with recent reports (Rausch et al., 2012). In summary, PDX models faithfully recapitulate important prognostic copy number alterations of pediatric tumors.

### Mutational Landscape of Models Derived from Tumors at Relapse

**Figure 3.**
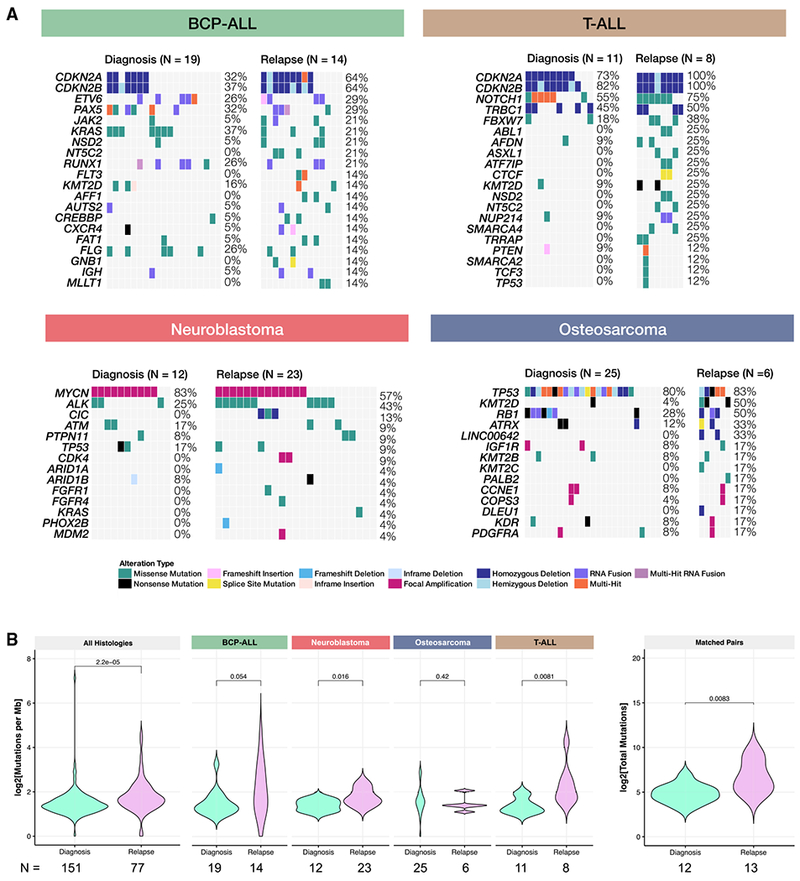
Mutational Landscape of Models Derived from Tumors at Relapse (A) For BCP-ALL, T-ALL, neuroblastoma, and osteosarcoma (histologies with N ≥ 6 models and multiple phases of therapy), oncoprints comparing hallmark alterations in models derived from diagnosis tumors to models derived from relapse tumors. (B) Tumor mutation burden (TMB) is significantly (or near significantly) higher in relapse models, compared to models established at diagnosis for all histologies collapsed (n_dx_ = 151, n_rel_ = 77, Wilcoxon p = 2.2e–5), BCP-ALL (n_dx_ = 19, n_rel_ = 14, Wilcoxon p = 0.051), neuroblastoma (n_dx_ = 12, n_rel_ = 23, Wilcoxon p = 0.016), and T-ALL (n_dx_ =11, n_rel_ = 8, Wilcoxon p = 0.0081). There was no difference between osteosarcoma models established at diagnosis and relapse (n_dx_ = 25, n_rel_ = 6, Wilcoxon p = 0.42). For patients in which models were established at both diagnosis and relapse, there was a significant increase in mutational burden upon relapse (n_dx_ = 12, n_rel_ = 13, p = 0.0083). All n’s denote biological replicates.

The majority of the PDX models were established at diagnosis (63%), but 6% were derived from surgical resection specimens after neoadjuvant therapy, 27% were from a relapsed specimen (14% of those were neuroblastomas from a large volume blood draw obtained immediately after death from disease progression), and 4% did not have the phase of therapy annotated. In addition, 12 pediatric cancer patients had either two or three models created across the spectrum of their therapy (Table S1). Here, we compare mutation frequencies and tumor mutation burdens (TMBs) for histologies with paired diagnosis-relapse cohorts with group N ≥ 6: BCP-ALL (N_diagnosis_ = 19/n_relapse_ = 14), T-ALL (n_diagnosis_ = 11/n_relapse_ = 8), osteosarcoma (n_diagnosis_ = 25/n_relapse_ = 6), and neuroblastoma (n_diagnosis_ = 12/n_relapse_ = 23). Across all four histologies, there is an increased frequency of key hallmark gene alterations in relapsed disease, as indicated by the oncoprint frequencies (Figure 3A). Using somatic missense and nonsense mutations, we calculated the TMB for each PDX model (STAR Methods). The median TMB across all models was 2.66 somatic mutations per megabase (Mut/Mb; Figure S3B; Table S3). The TMBs across this cohort of PDX models are likely higher than those in previous reports for two main reasons. First, 37% of the PDX models were derived from a patient tumor at a phase of therapy other than diagnosis, and it is now known that tumors acquire significantly more somatic mutations post-therapy and following a relapse (Eleveld et al., 2015; Ma et al., 2015; Padovan-Merhar et al., 2016; Schleiermacher et al., 2014; Schramm et al., 2015). Second, without paired normal samples, rare germline and private variants could not be reliably removed from the “somatic” MAF. Thus, the TMB reported here is likely inflated, but the trends across histologies and phase of therapy should accurately reflect TMBs determined with a paired germline sample. In fact, we observe an overall significantly higher TMB in PDX models derived from relapse tissue (3.08 Mut/Mb) compared to those derived at diagnosis (2.57 Mut/Mb, Wilcoxon p = 2.2e–5; Figure 3B). When compared to diagnostic tumors within a histology, the TMB was higher at relapse in BCP-ALL (Wilcoxon p = 0.054) and significantly higher at relapse in neuroblastoma (Wilcoxon p = 0.016) and T-ALL (Wilcoxon p = 0.0081), but it was not different between the diagnosis and relapse for osteosarcoma (Wilcoxon p = 0.42). Finally, we compared TMBs between paired diagnosis-relapse models and found a significantly higher TMB in models derived from relapse tumors (Figure 3B; median of 98.0 versus 27.5 mutations; Wilcoxon p = 0.0083). This PDX cohort recapitulates relapsed disease and provides a model for further studying tumor progression and therapeutic resistance.

### Expression Signatures Classify Pediatric PDX Models for TP53 and NF1 Inactivation

**Figure 4.**
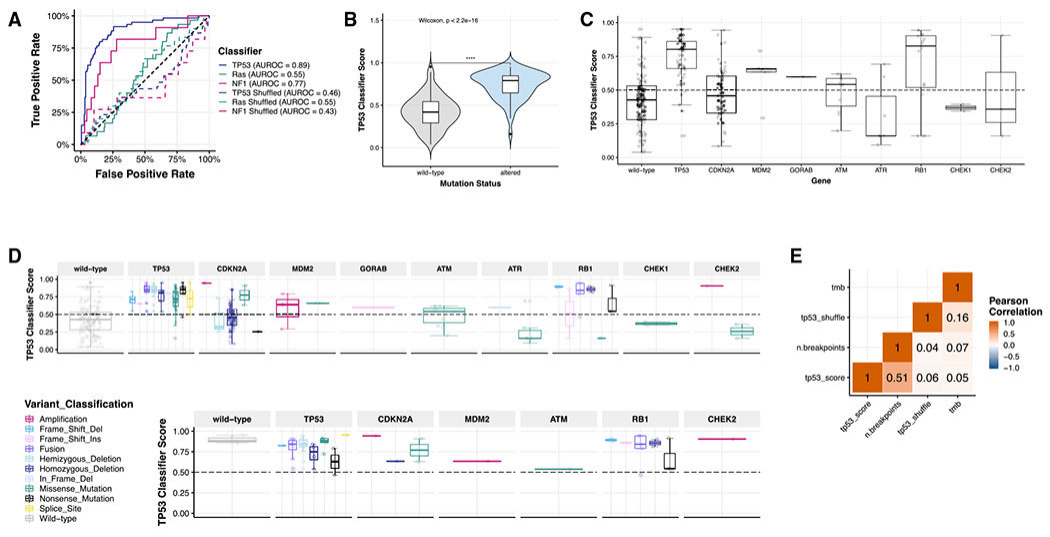
Expression and Mutational Signatures Classify Pediatric PDX Models for *TP53* and *NF1* Inactivation (A) Only *TP53* and *NF1* classifiers performed well in our dataset (AUROC_TP53_ = 0.89, AUROC_NF1_ = 0.77, AUROC_Ras_ = 0.55). Solid lines represent real scores, and dotted lines represent shuffled scores. Forthe samples measured (n = 244), 60 had *TP53* alterations (24.6%); 30 had *KRAS, HRAS*, or *NRAS* alterations (12.3%); and 11 had *NF1* alterations (4.5%). (B) *TP53* scores are significantly higher (n_WT_ = 120, n_ALT_ = 124, Wilcoxon p < 2.2 e–16) in models with genetic aberrations in *TP53* (mean score = 0.790) compared to those without alterations (mean score = 0.419). (C) Classifier scores are plotted based on the *TP53* pathway gene alteration present (n_WT_ = 120, n_TP53_ = 72, n_CDKN2A_ = 63, n_MDM2_ = 5, n_GORAB_ = 1, n_ATM_ = 11, n_ATR_ = 7, n_RB1_ = 16, n_CHEK1_ = 2, n_CHEK2_ = 3) or variant classification (n = 244 total samples). (D) *TP53* classifier scores across all histologies broken down by *TP53* pathway gene (n = 240). (E) In osteosarcoma models (n = 30), all scores, regardless of variant type or gene, were high and predicted pathway inactivation. Overall copy number burden (number of breakpoints calculated from SNP array data; STAR Methods) correlates significantly with *TP53* classifier score (R = 0.51, p = 1.8e–17, n = 239). All n’s denote biological replicates.

A recent study used TCGA data to classify tumors for *TP53* inactivation status and found that alterations in multiple genes phenocopy *TP53* inactivation, indicating that *TP53* mutation status alone is not necessary to infer the inactivation of the pathway (Knijnenburg et al., 2018). We applied a machine learning algorithm to infer *TP53* inactivation, *NF1* inactivation, and Ras pathway activation using PDX tumor transcriptomes. These classifiers were previously trained using gene expression data from TCGA PanCanAtlas (STAR Methods) (Knijnenburg et al., 2018; Way et al., 2017, 2018). The *TP53* (area under the receiver operator characteristic [AUROC] = 0.89) and *NF1* (AUROC = 0.77) classifiers are both accurate compared to a shuffled gene expression baseline, but performance of the Ras classifier (AUROC = 0.55) was relatively poor (Figure 4A), which may be attributed to differences in Ras pathway signatures in pediatric compared to adult tumors. Classifier scores >0.5 predict the inactivation of *TP53* or *NF1* (Table S5), and *TP53* scores are significantly higher (Wilcoxon p < 2.2e–16) in models with a *TP53* alteration (mean score = 0.790) compared to those without alterations (mean score = 0.419) (Figure 4B). Many models annotated as wild-type *TP53* have high *TP53* inactivation scores (Figure 4B). We found models with alterations in genes such as *MDM2* and *RB1* also have high *TP53* inactivation scores. These alterations may phenocopy *TP53* alterations (Figure 4C; genes chosen as primary or secondary interactors of *TP53* defined by the TP53 KEGG signaling pathway). In Figure 4D, we plot alterations for each gene by variant classification. Notably, all types of alterations within *TP53* were associated with high classifier scores, while the scores for other genes varied by type of alteration.

As *TP53* inactivation is a hallmark of osteosarcoma, we focused on these models as a proof of concept. The classifier predicted that all models profiled with RNA-seq except OS-55-SBX had *TP53* pathway inactivation. Many had a genetic alteration in a *TP53* pathway gene as supporting evidence (Figure 4E; Table S4). However, the mechanisms of *TP53* inactivation in OS-34-SJ, OS-43-TPMX, and OS-51-CHLX are still unknown and may require whole-genome sequencing to detect. To ensure osteosarcoma models were not driving the observed association with *TP53* scores, we removed the osteosarcoma models and reanalyzed the data. We found a significantly higher *TP53* classifier score (Wilcoxon p = 1.0e–11) in models with alterations in *TP53* pathway genes (Figures S5A and S5B). We then evaluated which types of variants were associated with high *TP53* classification scores and observed that models containing fusions had highest classifier scores compared to wild types, followed by models with single nucleotide variants (SNVs) and copy number variants (CNVs). (Figure S5C; Kruskal-Wallis p = 9.8e–11). These are broken down by gene in Figure S5D. Outside of osteosarcomas, only one model contained a fusion in the *TP53* pathway: Wilms model KT-9 contained a *TP53-FXR2* fusion. We found the overall copy number burden (number of breakpoints calculated from SNP array data; STAR Methods), but not the TMB or shuffle score, correlates significantly with the *TP53* classifier score (Figure 4E; R = 0.51, p = 1.8e–17), supporting recent published observations (Knijnenburg et al., 2018). Genetic alterations rendering *TP53* inactive may contribute to copy number instability in these models. The use of gene expression classifiers can guide preclinical studies: for example, therapeutically targeting the *TP53* pathway in tumors with high *TP53* inactivation scores rather than those with altered *TP53*.

#### Expression Profiles of PDX Models Cluster by Tissue of Origin and Contain Driver Fusions

**Figure 5.**
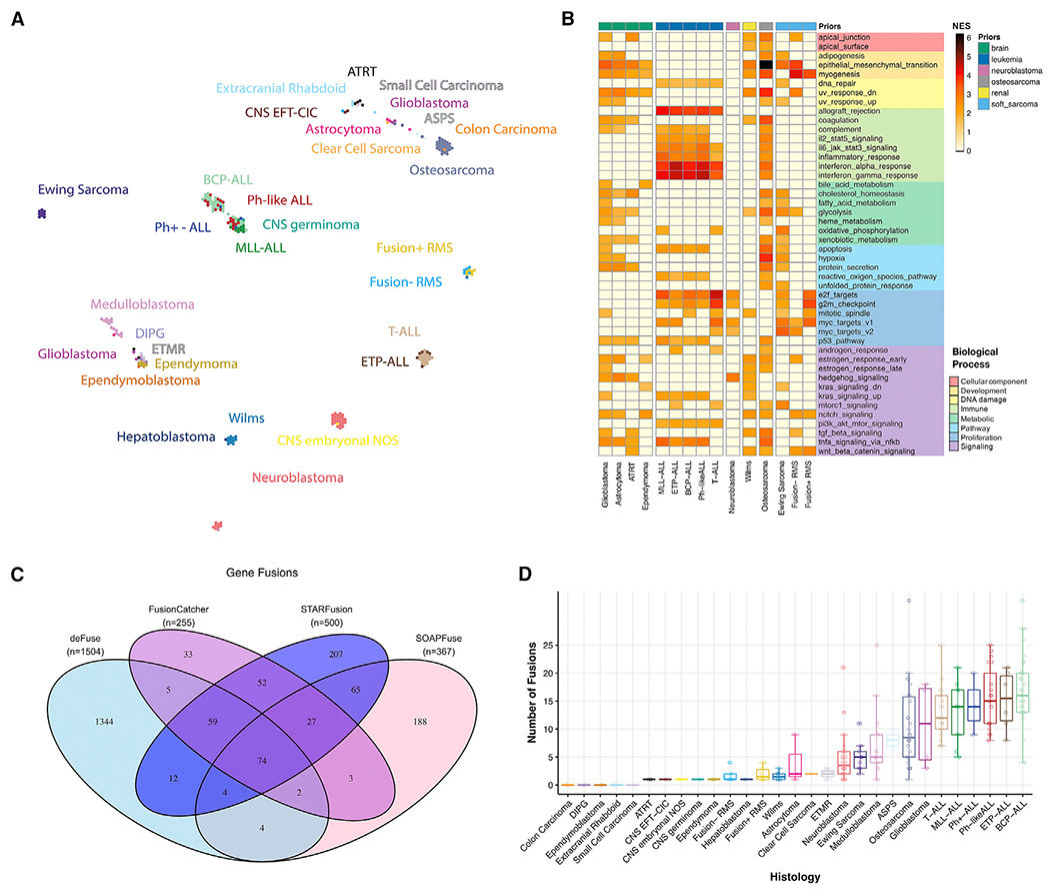
Expression Profiles of PDX Models Cluster by Histology and Contain Driver Fusions (A) TumorMap rendition of PDX RNA-seq expression matrices by histology. (B) Gene set enrichment analysis for Hallmark pathways for histologies with n ≥ 4 samples demonstrates histology-specific biologic processes significantly altered (adjusted p < 0.05 and NES > 2.0, N = 221). Samples were grouped by prior before GSEA (n_bone sarcoma_ = 10, n_brain_ = 58, n_leukemia_ = 90, n_neuroblastoma_ = 35, n_osteosarcoma_ = 36, n_renal_ = 14, n_soft sarcoma_ = 18). (C and D) Venn diagram of RNA fusion overlap among four algorithms (C) and high-confidence fusion totals (D) demonstrates a higher overall number of fusions in hematologic malignancies (boxplots are graphed as medians with box edges as first and third quartiles; detailed Ns in Table S3). n = 244 RNA samples used as input, and all n’s represent biological replicates.

We used the UCSC TumorMap (Newton et al., 2017) to visualize clusters of expression profiles across PDX histologies (Figure 5A). We observed a clear separation among unrelated histologies and an overlapping clustering among related histologies. For example, T-ALL and ETP-ALL cluster together as expected, but distinctly from other ALL histologies. The leukemias clustered by subtype and distinctly from solid tumors. Ewing sarcoma, neuroblastoma, Wilms, and medulloblastoma form distinct clusters. Osteosarcomas cluster with two ASPS models. Fusion+ and Fusion-RMS cluster near each other but distinctly. Brain tumor histologies cluster near each other with the exception of ATRTs, some of which cluster with extracranial rhabdoid tumors near sarcoma samples. We identified histology-specific expression differences using a Bayesian hierarchical model (Gelman, 2006), grouped related histologies under the same prior distribution, ranked gene expression differences for each histology, and performed GSEA. This demonstrated tissue-specific enrichment within each histology, using GSEA and the Tissue-Specific Gene Database in Cancer (TissGDB; Kim et al., 2018a) and Tissue-Specific Gene Expression and Regulation Database (TiGER; Liu et al., 2008) gene sets (Figure S5F). To investigate pathway enrichment within histologies, we ran GSEA using the MSigDB curated (C2) gene sets and plotted the normalized enrichment scores (NESs) for the Hallmark pathway gene sets in Figure 5B.

Next, we created a high-confidence fusion annotation pipeline (Figure S1; STAR Methods) using four algorithms: defuse, FusionCatcher, STARFusion, and SOAPFuse. A total of 50,796 unique fusions were called, and we defined 925 unique high-confidence fusions and 92 unique known oncogenic driver fusions defined by cytogenetics and literature (Figure 5C; Table S5). Fusions were annotated for their frame and for whether a gene partner is a known oncogene, kinase, or transcription factor to identify oncogenic potential and functional relevance. We found that PPTC PDX models largely maintain known oncogenic driver fusions specific to their histologies: all alveolar rhabdomyosarcoma models harbored *PAX3-FOXO1* fusions, all Ewing sarcoma samples with RNA-seq data showed *EWSR1-FLI1* fusions, all Ph+ ALL tumors contained *BCR-ABL1* fusions, and *KMT2A* (*MLL*) fusions were detected in all MLL-ALL models (Table S5). Osteosarcomas harbored *TP53* fusions, and breakpoints reside within intron one of the *TP53* gene, a mechanism of TP53 inactivation previously reported in osteosarcoma (Ribi et al., 2015). In five diagnosis-relapse pairs, we detected four fusions in the diagnostic PDX (*PAX5-RP11-465M18.1*, *IGH-MYC, CIC-DUX4*, and *TP53-TNR*) that were undetected in their paired relapse model, suggesting these specific gene fusions may have been acquired after an alternative initiating event that was retained.

## DISCUSSION

Here, we used whole-exome, whole-transcriptome, SNP genotyping arrays, and STR profiling to characterize 261 pediatric PDX models across 37 unique molecular subtypes. We used a competitive mapping approach to remove mouse reads from DNA or RNA-seq data and demonstrated high concordance between these pipelines and the orthogonal measurement of human:mouse DNA ratios. We showed a faithful recapitulation of primary and relapsed disease within tumor of origin type through analysis of somatic mutations, copy number alterations, RNA expression, gene fusions, and oncogenic pathways. It is clear that the models here are biased toward the most highly aggressive pediatric cancers, which is reflective of the typical pediatric phase 1 patient populations.

The data presented herein have immediate applications to the prioritization of experimental agents for testing in pediatric preclinical models, leading to eventual clinical testing. For example, there are reports identifying specific genomic alterations as predicting sensitivity to ATR inhibitors, including ATM loss, ARID1A mutation, defective homologous recombination, and ATRX mutation associated with alternative lengthening of telomeres (ALTs) (Lecona and Fernandez-Capetillo, 2018). Querying the PPTC data at PedcBioPortal can quickly identify models with these characteristics, and the models can then be used to test whether *in vivo* responsiveness to ATR inhibitors is predicted by one or more of the molecular characteristics. Similarly, PPTC RNA-seq data can be used to identify models that show elevated gene expression for the targets of immunotherapeutics such as antibody-drug conjugates and T-cell engagers. As examples, in the PPTC dataset, GPC2 and ALK are dramatically overexpressed neuroblastoma models, as previously published (Bosse et al., 2017; Sano et al., 2019), but also in multiple subsets of additional pediatric cancer histotypes, allowing for a basket trial design for preclinical testing. The PPTC RNA-seq dataset was also used to identify T-ALL as a target histology for an agent activated by the aldo-keto reductase AKR1C3 (R.B. Lock et al., 2018, Mol. Cancer Ther., abstract) and to identify ASPS xenografts as intrinsically overexpressing CD274 (PD-L1), making ASPS a target histology for the evaluation of checkpoint inhibition (C.G. O’Sullivan et al., 2018, Connective Tissue Oncology Society Annual Meeting, conference).

Further, we performed machine learning to classify tumors into TP53 and NF1 active or inactive, and we suggest that these scores might be future biomarkers for drug response. These classifiers have been used to identify tumors that may respond to novel agents, including those that target tumors driven by NF1 loss (Way et al., 2017). Although these machine learning algorithms are not ready for the clinic, the next logical step is to use PDX models to test the predictive nature of classifiers so that in the future, interdisciplinary teams can identify tumors driven by TP53 and/or NF1 loss, evaluate, and compare multiple therapies in real time.

Our study also highlights additional opportunities for pan-pediatric genomic characterization. We did not have available models for acute myelogenous leukemia, juvenile myelomonocytic leukemia, lymphomas, retinoblastoma, melanoma, thyroid malignancies, or histone mutant midline gliomas. Additionally, although we covered 37 molecular subtypes, many of the rare tumors had low numbers of models and could benefit from the creation and sequencing of additional PDXs, and we seek to generate these data and/or hope to merge our data with future pediatric cancer PDX sequencing projects. Finally, WES likely missed several pathogenic lesions, and DNA methylation profiling is particularly relevant for pediatric brain tumors. Future studies, perhaps in collaboration with ongoing similar efforts by international colleagues, could address these gaps.

We performed this project to provide a resource to the pediatric cancer research community. To date, the pediatric cancer genomic literature largely focuses on diagnostic samples, and this study includes a large number of PDXs derived during or after intensive chemoradiotherapy. Thus, the frequency of many genomic alterations is higher in these models compared to the literature. By having a large number of PDXs obtained from samples at relapse or at autopsy, we can provide models that more closely recapitulate the patients being enrolled in early-phase clinical trials after extensive chemoradiotherapy. All models and data are freely available for the cancer research community, as described in the STAR Methods.

## STAR★METHODS

### LEAD CONTACT AND MATERIALS AVAILABILITY

Further information and requests for resources and reagents should be directed to, and will be fulfilled by, the Lead Contact, John M. Maris (maris@email.chop.edu). All PDX models are available through the Pediatric Preclinical Testing Consortium with a completed Material Transfer Agreement.

### EXPERIMENTAL MODEL AND SUBJECT DETAILS

#### Patient-Derived Xenograft Generation and Harvesting

Patient-derived xenograft models from the Pediatric Preclinical Testing Program (PPTP) were generated as described (Houghton et al., 2002, 2007; Whiteford et al., 2007). Briefly, for solid tumors, C.B-Igh-1b/IcrTac-Prkdcscid (Taconic Farms, Germantown NY), were subcutaneously flank-engrafted into male or female mice (Table S1) and passaged once tumors reached 200 mm^3^. For CNS tumors, patient tumors were stereotactically-transplanted into anesthetized (50 mg/kg sodium pentobarbital) RAG2, NOD.129S7(B6)-Rag1tm1Mom/J, or RAG1tm1Mom/J mouse brains in the diagnosis-specific orthotopic locations noted in Table S1 (Yu et al., 2010). PDX tumor cells (1 × 10^5^) were suspended in 2 ul of culture media and slowly injected through a burr hole using a 10 ul, 26 gauge syringe into the brain region of interest. Once moribund, or displaying neurological deficit symptoms, mice were euthanized and whole murine brains containing visible tumors were aseptically removed and transferred to the tissue culture laboratory. Tumors were microscopically dissected from surrounding brain tissue, mechanically dissociated into cell suspensions, and filtered. Single tumor cells were subsequently injected into the brains of SCID mice as described above. Sub-transplantation process was repeated to complete a total of five tumor passages. All animal experiments were conducted according to an Institutional Animal Care and Use Committee-approved protocol. All leukemia animal experimentation was approved by the Animal Care and Ethics Committee, UNSW Sydney (Sydney, Australia). Experiments used continuous PDXs established previously in 20-25 g female non-obese diabetic/severe combined immuno-deficient (NOD.CB17-Prkdc^scid^/SzJ, NOD/SCID) or NOD/SCID/interleukin-2 receptor *γ*-negative (NOD.Cg-Prkdc^scid^ Il2rg^tm1Wjl^/SzJ, NSG) mice. Leukemia cells were inoculated intravenously into 6-8 week-old NOD/SCID or NSG mice (Australian BioResources, Moss Vale, NSW, Australia) and leukemia burden monitored via enumeration of human CD45^+^ (%huCD45^+^) cells versus total CD45^+^ leukocytes (human plus mouse) in the peripheral blood (PB) and tissues, as reported (Liem et al., 2004; Lock et al., 2002). The continuation of xenograft lines was accomplished through harvesting human leukemia cells from the spleens of the engrafted mice. Harvesting required more than 3 × 10^8^ leukemia cells per spleen, at 85% purity. Additional details per model including sex, age, and mass are included in Table S1.

### METHOD DETAILS

#### Nucleic Acid Extractions and Quality Control

PDX samples were submitted from Children’s Cancer Institute, Children’s Hospital of Philadelphia, Greehey Children’s Cancer Research Institute, and Montefiore Medical Center to the Nationwide Children’s Hospital Biospecimen Core Resource at −190°C using an MVE cryoshipper. Cytospins and H&E frozen sections were prepared from leukemia and solid tissue PDX specimens, respectively. Slides were assessed by board-certified pathologists to determine blast percentage in leukemia PDX samples, and percent tumor nuclei and necrosis of the solid PDX samples. DNA and RNA were co-extracted from the PDXs using a modification of the DNA/RNA AllPrep kit (QIAGEN). The flow-through from the QIAGEN DNA column was processed using a mirVana miRNA Isolation Kit (Ambion). DNA was quantified by PicoGreen assay and RNA samples were quantified by measuring Abs_260_ with a UV spectrophotometer. DNA specimens were resolved by 1% agarose gel electrophoresis to confirm high molecular weight fragments. RNA was analyzed via the RNA6000 Nano assay (Agilent) for determination of an RNA Integrity Number (RIN). The PPTC study committee reviewed the pathology and molecular QC data and selected DNA and RNA aliquots for sequencing.

#### Short Tandem Repeat (STR) Profiling

Each tumor DNA sample was subjected to STR profiling performed by Guardian Forensic Sciences. DNA samples were quantified using QIAGEN Investigator Quantiplex Kit (Cat# 387018) on a QIAGEN RotorGene Q instrument. The GenePrint24 System for STR profiling (Promega, Cat#B1870) was used to amplify 0.05 ng of template DNA in a 12.5 μL volume using the following conditions: 96°C for 1 minute, 27 cycles of {94°C for 10 s, 59°C for 1 minute, 72°C for 30 s}, 60°C for 10 minutes using the RotorGene Q instrument. Samples were injected into the Applied Biosystems ABI 310 Genetic Analyzer and profiles were interpreted by forensic biologists. Only those samples deemed not misidentified and free of contamination were used in this study.

#### Biochemical Measurement of Human DNA Content in PDX Tumors

To determine the composition of human and mouse DNA within PDX tumors, PDX DNA samples were amplified using modified version of the published *pTGER2* (*prostaglandin E receptor 2*) qPCR assay (Alcoser et al., 2011). Depending upon sample availability, 2-20 ng of PDX tumor DNA were added to 500 nM each human- and mouse-specific forward primers, reverse primers, probes (sequences in resource document) and 1X IDT PrimeTime Gene Expression 2X Mastermix (Integrated DNA Technologies) in a total of 20 uL. Reactions were thermalcycled at 95°Cfor 8 min and 42 cycles of {95°Cfor 15 s, 64°C for 1 min}. Five-point standard curves were performed using a mixture of CHLA-90 and COG-N-603 neuroblastoma cell lines as human-specific template and pooled liver/spleen/muscle DNA from a naive NU/NU mouse as the mouse-specific template to confirm each primer efficiency was between 90%–110%. The DNA equivalent of one diploid copy of either mouse or human template was run as a reference template. Three technical replicates were performed for each standard and sample. Average C_T_ values of the reference DNA samples were used as “ground truth” C_T_ values for one DNA copy. To estimate relative copy number, 2^−ΔCT^ values were calculated for each unknown for each species: 2^−Δ*CT*^ = 2^−(*CT of Unknown*−*CT of Refererence*)^. To estimate percent human content, the following equation was used: %*Human content* = (*Relative human genome copies* × 100/*Relative mouse genome copies*).

#### Additional Quality Control for Cross-Contamination and Mis-Identification

Common germline SNP distributions (allele frequency > 0.005 in any one of the three databases: Exome Aggregation Consortium, 1000 genomes, or the NHBLI Exome Sequencing Project) were plotted for each model and visually inspected for a negatively skewed distribution to assess DNA cross-contamination in WES data. To identify potential mis-identification, RNA variant calling was performed and variant allele frequencies correlated between WES and RNA. Models whose variants did not correlate were deemed mis-identified and removed (STAR Methods). For remaining models, NGScheckmate was performed between WES and RNA data. All models except for ICb-2002EPN had correlation values of ≥ 0.61 at depths of ≥ 10, deeming these models matched as recommended by Lee et al. (2017). ICb-2002EPN had a borderline correlation of 0.6025 at a depth of 14.51, but deemed matched from WES-RNA mutation correlations. Within this cohort, five pairs of models were derived from tissue at phase of therapy (Table S1). Thus, as additional QC, we correlated somatic mutation allele frequencies between each pair and found high concordance of mutation frequencies (data on Figshare, STAR Methods), confirming biological reproducibility of creating PDX models within a center. Mutation variation is summarized per model in Table S3.

#### Whole Exome Sequencing

Illumina paired-end pre-capture libraries were constructed from PDX DNA samples according to the manufacturer’s protocol (Illumina Multiplexing_SamplePrep_Guide_1005361_D) modified as described in the BCM-HGSC Illumina Barcoded Paired-End Capture Library Preparation protocol. The complete protocol including oligonucleotide sequences used as adaptors and blockers are accessible from the HGSC website https://www.hgsc.bcm.edu/sites/default/files/documents/Protocol-Illumina_Whole_Exome_Sequencing_Library_Preparation-KAPA_Version_BCM-HGSC_RD_03-20-2014.pdf. The DNA sequence production is briefly described below.

##### Library Preparation

500 ng (or 250 ng if sample quantity was limiting) of DNA in 50ul volume were sheared into fragments to an average size of 200-300 bp in a Covaris plate with E220 system (Covaris, Inc. Woburn, MA) followed by end-repair, A-tailing and ligation of the Illumina multiplexing PE adaptors. Pre-capture Ligation Mediated-PCR (LM-PCR) was performed for 6-8 cycles using the Library Amplification Readymix containing KAPA HiFi DNA Polymerase (Kapa Biosystems, Inc.). Universal primer LM-PCR Primer 1.0 and LM-PCR Primer 2.0 were used to amplify the ligated products. Reaction products were purified using 1.8X Agencourt AMPure XP beads (Beckman Coulter) after each enzymatic reaction. Following the final 1.2X Agencourt XP beads purification, quantification and size distribution of the pre-capture LM-PCR product was determined using Fragment Analyzer capillary electrophoresis system (Advanced Analytical Technologies, Inc.).

##### Capture Enrichment

Four pre-capture libraries were pooled together (~750 ng/sample, 3 ug/pool) and then hybridized in solution to the HGSC VCRome 2.1 design1 (Bainbridge et al., 2011) according to the manufacturer’s protocol NimbleGen SeqCap EZ Exome Library SR User’s Guide (Version 2.2) with minor revisions. Probes for exome coverage across > 3,500 clinically relevant genes that are previously < 20X (~2.72Mb) is supplemented into the VCRome 2.1 probe. Human COT1 DNA was added into the hybridization to block repetitive genomic sequences. Blocking oligonucleotides from Sigma (individually sequence specifically synthesized) or xGen Universal Blocking oligonucleotides (Integrated DNA Technologies) were added into the hybridization to block the adaptor sequences. Hybridization was carried out at 560C for ~16h. Post-capture LM-PCR amplification was performed using the Library Amplification Readymix containing KAPA HiFi DNA Polymerase (Kapa Biosystems, Inc.) with 12 cycles of amplification. After the final AMPure XP bead purification, quantity and size of the capture library was analyzed using the Agilent Bioanalyzer 2100 DNA Chip 7500. The efficiency of the capture was evaluated by performing a qPCR-based quality check on the four standard NimbleGen internal controls. Successful enrichment of the capture libraries was estimated to range from a 6 to 9 of ΔC_T_ value over the non-enriched samples.

##### DNA Sequencing

Library templates were prepared for sequencing using Illumina’s cBot cluster generation system with TruSeq PE Cluster Generation Kits (Illumina) according to the manufacturer’s protocol. Briefly, these libraries were denatured with sodium hydroxide and diluted to 6-9 pM in hybridization buffer in order to achieve a load density of ~800K clusters/mm^2^. Each library pool was loaded in a single lane of a HiSeq flow cell, and each lane was spiked with 1% phiX control library for run quality control. The sample libraries then underwent bridge amplification to form clonal clusters, followed by hybridization with the sequencing primer. Sequencing runs were performed in paired-end mode using the Illumina HiSeq 2000 platform. Using the TruSeq SBS Kits (Illumina), sequencing-by-synthesis reactions were extended for 101 cycles from each end, with an additional 7 cycles for the index read. With sequencing yields averaging 12.1 Gb per sample, samples achieved an average of 97.64% of the targeted exome bases covered to a depth of 20X or greater.

##### Primary Data Analysis

Initial sequence analysis was performed using the HGSC Mercury analysis pipeline (Challis et al., 2012; Reid et al., 2014). In summary, the. bcl files produced on-instrument were first transferred into the HGSC analysis infrastructure by the HiSeq Real-time Analysis module. Mercury then ran the vendor’s primary analysis software (CASAVA) to de-multiplex pooled samples and generate sequence reads and base-call confidence values (qualities), followed by the mapping of reads to the GRCh37 Human reference genome (https://www.ncbi.nlm.nih.gov/projects/genome/assembly/grc/human/) using the Burrows-Wheeler aligner (Li and Durbin, 2010). The resulting BAM (binary alignment/map) file underwent quality recalibration using GATK, and where necessary the merging of separate sequence-event BAMs into a single sample-level BAM. BAM sorting, duplicate read marking, and realignment to improve in/del discovery all occur at this step. Next, Atlas-SNP and Atlas-indel from the Atlas2 suite (Shen et al., 2010) were used to call variants and produce a variant call file (VCF). Finally, annotation data was added to the VCF using a suite of annotation tools “Cassandra” (https://www.hgsc.bcm.edu/software/cassandra) that brings together frequency, function, and other relevant information using AnnoVar with UCSC and RefSeq gene models, as well as a host of other internal and external data resources.

#### SNP Array Assay

In brief, 200 ng of genomic DNA were denatured with NaOH, followed by isothermal whole genome amplification at 37°C for 20-24 hours. The amplified DNA was enzymatically fragmented and hybridized to the BeadChip for 16-24 hours at 48°C (24 samples were processed in parallel for each BeadChip). After a series of washing steps to remove unhybridized and non-specifically hybridized DNA fragments, allele-specific single-base extension reactions were performed to incorporate labeled nucleotides into the bead-bound primers. A multi-layer staining process was conducted to amplify signals from the labeled extended primers, and then the coated beads were imaged with the Illumina iScan system.

Chip types used were humanomniexpress-24-v1-1-a.bpm and InfiniumOmniExpress-24v1-2_A1.bpm.

#### Whole Transcriptome Sequencing

Whole-transcriptome RNA sequencing (RNA-seq) was performed using total RNA extracted as described above. Strand-specific, poly-A+ RNA-seq libraries for sequencing on the Illumina platform were prepared using manufacturer guidelines with minor modifications described herein (Peters et al., 2015; Wang et al., 2015). RNA Integrity was confirmed (RIN > 7.0) on a Bioanalyzer (Agilent). Briefly, poly-A+ mRNA was extracted from 1 μg total RNA using Oligo(dT)25 Dynabeads (Life Technologies), to which 4 μL of 1:100 dilution of the ERCC spike-in mix 1 (Ambion, Life technologies) was already added (Baker et al., 2005). There are a total of 92 polyadenylated transcripts in this mix that are used to monitor sample and process consistency. mRNA is then fragmented by heat at 94°C for 15 minutes or less depending on sample RIN. First strand cDNA was synthesized using NEBNext RNA First Strand Synthesis Module (New England BioLabs) and during second strand cDNA synthesis, dNTP mix containing dUTP was used to introduce strand-specificity with NEBNext Ultra Directional RNA Second Strand Synthesis Module (New England BioLabs). For Illumina paired-end library construction, the resultant cDNA is processed through end-repair and A-tailing, ligated with Illumina PE adapters, and then digested with 10 units of Uracil-DNA Glycosylase (New England BioLabs). Libraries are prepared on the Beckman BioMek FXp robots and amplification of the libraries was performed for 13 PCR cycles using the Phusion High-Fidelity PCR Master Mix (New England BioLabs); 6-bp molecular barcodes that were also incorporated during this step. Libraries were purified with Agencourt AMPure XP beads (Beckman Coulter) after each enzymatic reaction, and after PCR amplification, and were quantified using Fragment Analyzer electrophoresis system. Libraries were pooled in equimolar amounts (4 libraries/pool). Library templates were prepared and sequenced exactly as described above for DNA Sequencing. Sequencing runs generated approximately 300-400 million successful reads on each lane of a flow cell, yielding 75-100M reads per sample.

### QUANTIFICATION AND STATISTICAL ANALYSIS

#### Mouse Read Subtraction from WES Sequencing Data

Raw fastq files (n = 240) from Whole exome sequencing data were aligned to a combined hybrid genome of human hg19 and mouse mm10 genomes using the *Burrows-Wheeler transformation algorithm (BWA v0.7.17-r1188)*. Reads overlapping specifically to either the human or mouse genome were extracted and separated in corresponding human and mouse bam files using Samtools v1.9. The mouse subtracted bam files containing reads specific to human genome were then sorted by name and only paired reads were kept using the Samtools parameter −*f 1*. Following this, duplicated reads were marked using Sambamba v0.6.6. The resulting bam files were then used as input for local realignment around indels using IndelRealigner and base quality score recalibration using BaseRecalibrator utilities from GATK v3.8.1.

#### Whole Exome Mutation Analysis

Many of these PDX models have been established decades ago, thus matched primary and/or normal tissue either were not collected or is not currently available. To filter common germline variation from these tumor models, we used a panel of 809 normal samples supplied from TCGA WBC tissue to generate consensus germline variant calls. Rare germline variation was retained and defined as < 0.005 minor allele frequency in any one of the three databases: Exome Aggregation Consortium (ExAC) (Lek et al., 2016), 1000 genomes, or the NHBLI Exome Sequencing Project (ESP). Filtered variants also present in COSMIC were scavenged back. We performed MutSigCV (Lawrence et al., 2013) analysis on the entire cohort to identify and remove false positive variants. With the exception of known oncogenes and tumor suppressors, novel significantly mutated genes (SMGs) common across all histologies should be rare. We manually inspected the top 100 SMGs and found that most novel genes harbored a high number of private mutations and thus were not removed. Other novel variants were false positives due to germline inclusion or sequencing/mapping errors (data on FigShare, link below). Data were thus split into germline MAF and somatic MAF files, the latter of which retained private variants.

#### Tumor Mutation Burden Analysis

Using the maftools R package (Mayakonda et al., 2018), total number of mutations per variant type per model were calculated. We defined tumor mutation burden using only mononucleotide substitutions resulting in amino acid changes: (*Σ*(*somatic nonsynonymous* + *missense variants*)/45.1 *Mb*). The denominator was the 45.1 Mb size of the Roche Nimblegen VCRome v. 2.1 capture panel.

#### ATRX Deletion Analysis

The *ATRX* locus on chromosome X contains too few probes in OmniExpress arrays to accurately assess deletion, even in cases of known sex. Thus, from WES bam files, total read base counts for ATRX exons were calculated using Samtools v1.9 bedcov utility and total library size was calculated using Samtools v1.9 flagstat utility. To convert exon read counts to Fragments per kilobase per million reads (FPKM), the library sizes were first transformed to per million scaling factors. Following this, raw read counts of each exon were normalized using the per million scaling factors and the corresponding exon length.

#### Mutational Signatures Analysis

The deconstructSigs R package with the COSMIC 30 signature reference was used. We ran this workflow on models with ≥ 50 total somatic mutations. We chose a cosine similarity value cutoff at 0.1 and plotted the proportion of signatures in each model as a stacked barplot.

#### Classifier Analysis

We applied models derived from three supervised machine learning algorithms to all PDX models with available RNA-Seq data (n = 244). The models were previously trained on RNaseq, copy number, and mutation data across 33 different adult cancer-types from The Cancer Genome Atlas PanCanAtlas project (Cancer Genome Atlas Research Network et al., 2013). Briefly, the algorithm was an elastic net penalized logistic regression classifier that took FPKM and z-score normalized RNaseq data as input and, in three independent classifiers, was trained to predict Ras pathway activation, *NF1* inactivation, and *TP53* inactivation using mutation and copy number alteration status of corresponding samples. The Ras pathway and *NF1* classifiers and the overall method were described in more detail in Way et al. (2018). The application and validation of the *TP53* classifier was described in Knijnenburg et al. (2018).

To assess performance of the TCGA trained classifiers applied to the PDX data, we used orthogonal evidence of gene alterations in each PDX sample. Specifically, we used samples with observed missense, nonsense, frameshift, and splice site mutations in *ALK, BRAF, CIC, DMD, HRAS, KRAS, NF1*, *NRAS, PTPN11*, and *SOS1* as samples with possible Ras pathway activation. We used samples with only non-silent *NF1* mutations for the *NF1* classifier, and samples with deleterious *TP53* mutations, copy number deletions, and fusions for the *TP53* classifier. We assessed model performance using receiver operating characteristic (ROC) and precision recall (PR) curves using these samples as the positive set and all others as the negative set. We also applied the classifiers to shuffled PDX gene expression matrices and compared performance to the real data to assess potential model bias. The reproducible analysis pipeline can be viewed at https://github.com/marislab/pdx-classification and the software is archived on Zenodo at https://doi.org/10.5281/Zenodo.1475249.

#### mRNA Gene Expression Analysis

Raw fastq files (n = 244) from RNA-sequencing data were aligned to a combined hybrid genome of human hg19 and mouse mm10 genomes using the STAR aligner v2.5.3a. Reads overlapping specifically to either the human or mouse genome were extracted and separated in corresponding human and mouse bam files using Samtools v1.9. The mouse subtracted bam files containing reads specific to human genome were then sorted by name and only paired reads were kept using the Samtools parameter −*f 1*. Following this, duplicated reads were marked using Sambamba v0.6.6. The resulting bam files were used to extract and separate reads into paired-ended fastq files using the *SamToFastq* utility of Picard v2.18.14-0. The resulting paired-ended fastq files obtained after mouse subtraction were re-aligned to human genome hg19 using STAR aligner and marked for duplicate reads using Picard *MarkDuplicates*. Gene expression was quantified in terms of Fragments Per Kilobase of transcript per Million mapped reads (FPKM) using HTSeq v0.9.1 and Cufflinks v2.2.1. We also processed RNA-sequencing patient data from TARGET (ALL, n = 533; AML, n = 364; NBL, n = 169; RT, n = 70; OS, n = 87; WT, n = 136) and PPTC PDX data (n = 244) using STAR alignment and RSEM normalization using hg38 as reference genome and Gencode v23 gene annotation to get transcript per million (TPM) expression values. For PPTC PDX data, human bam files generated from the mouse subtraction pipeline were used in order to generate input fastq files.

#### mRNA Variant Calling, Filtering, and Comparison to DNA Variants

Variant calling for RNA-seq samples was performed with Strelka v2.9.2 germline indels calling pipeline using hg19 primary assembly reference fasta and default parameters. VCFs were converted to MAF and variants were filtered for those that passed VEP and were non-silent (! = Silent or Intron). Variant allele frequencies for all non-silent, VEP-passed RNA variants were calculated. For each model on which both WES and RNA-Seq were performed, WES variants with RNA evidence were matched in the DNA MAF and VAF correlations were plotted and are stored in the QC folder of the FigShare project: https://figshare.com/projects/Genomic_profiling_of_childhood_tumor_patient-derived_xenograft_models_to_enable_rational_clinical_trial_design/38147.

#### Copy Number Analysis

SNP arrays were processed at the HGSC using the Illumina Infinium HTS Assay according to the manufacturer’s guidelines. Human OmniExpress arrays (Illumina, catalog No. WG-315-1101) were used, interrogating 741 thousand SNP loci with a MAF detection limit of 5%. SNP calls were collected using Illumina’s GenomeStudio software (version 1.0/2.0) in which standard SNP clustering and genotyping were performed with the default settings recommended by the manufacturer. Data from samples that met a minimum SNP call rate of 0.9 were considered passing and were included in subsequent analyses. Output files from Genome Studio containing BAF and LRR were used as input for Nexus 8.0. Quadratic systematic correction was performed using a custom file (Figshare repository, below) containing common snp probes from the two chip types. The significance threshold was reduced to 1 × 10^−8^ to reduce background noise. Segmentation was performed using Nexus’s SNPRANK algorithm. To extract segments, gain was set to 0 and loss to −1 × 10^−11^. The output table was reformatted to segmentation file format for input to GISTIC2.0, which was used to calculate broad and focal, hemizygous gene-level copy number events. Relevant arm and band level alterations were used in oncoprints. Since normal DNA was not available for paired analyses, sex chromosomes were removed. Focal homozygous deletions and amplifications were annotated using the segmentation file created post-Nexus analysis. A cutoff of LRR > = (0.538578182) was used for amplifications and > = (−1.739) for deletions. Cutoffs were determined by assessing histogram splits for MYCN amplification, SMARCB1 deletion, and CDKN2A/B deletions. Homozygous deletions remained only if mRNA FPKM was < 5 or if RNA-Seq for a sample was not available. Manual inspections were performed to confirm alterations for *SMARCB1, TP53, WT1, MYCN, C19MC, CDKN2A/B* and edited when necessary (see code).

#### Breakpoint Analysis

We defined breakpoint regions as regions with 10% copy number change between adjacent segments. These were tabulated per autosome per model and plotted by histology in Figure S4C. To defined regions of high breakpoint density (HBD) as ≥ 10 breakpoints per chromosome (Figure S4D; Table S3).

#### Ethnicity Inference

Approximate genomic ancestries for each PDX model were inferred through principal component analysis of SNP array genotypes. Illumina-designated plus-strand genotypes were exported from GenomeStudio and processed using PLINK 1.9. Sex chromosomes and SNPs with minor allele frequency < 1%, call rate < 90%, ora deviation from Hardy-Weinberg equilibrium surpassing p = 0.00005 were excluded. The PDX dataset was then merged with HapMap 3 (draft release 2), restricting to only the intersecting SNPs. This set was pruned to remove highly correlated SNPs using a window size of 50 variants, step size of 5 variants, and pairwise r^2^ threshold of 0.1. The 39,544 remaining SNPs were used to calculate the top 20 principal components. Approximate ethnicities were inferred using the first two components. Individuals were classified into four broad population groups: European (including HapMap CEU and TSI population samples), African (ASW, LWK, MKK, and YRI), East Asian (CHB, CHD, and JPT), and South Asian or Hispanic (GIH and MXL).

#### Fusion Transcript Analysis

We used four different fusion callers: STAR-Fusion v1.1.0, FusionCatcher v0.99.7b, deFuse and SOAPFuseon RNA-sequencing data of the PDX models (n = 244). A total of 50,796 unique fusions were predicted with the following breakdown: STAR-Fusion (n = 9,496), FusionCatcher (n = 3,822), deFuse (n = 30,393), and SOAPFuse (n = 7,085). To reduce the number of false positives, we used two parallel approaches: first to keep all fusions predicted as in-frame and second to keep all fusions where the 5′ or 3′ gene fuses promiscuously with multiple partners within the same histology. To filter out unreliable predictions, we further filtered the in-frame fusions by keeping fusions that were recurrently predicted in two or more models within the sample histology or fusions that were supported by at least two fusion callers. We removed any fusions where expression of both genes in the gene pair was found to be < 1 TPM value across all models or it was not reported by the gene quantification algorithm. We then combined the lists from the two approaches discussed above and filtered out any fusions that were predicted in more than one histology. To remove spurious fusions, we filtered all fusions annotated as “read-through” as a result of fusions between adjacent or neighboring genes. We further removed fusions identified in non-cancer tissues and cells as per GTEx in order to remove chimeric RNA that is normally found in healthy tissue. Next, we scavenged and annotated fusions that have been identified as “driver” fusions in literature and fusions that were validated using cytogenetics. Finally, we annotated the gene fusion partners with oncogenes from COSMIC, kinases from Kinase.com, and transcription factors from AnimalTFDB to identify any oncogenic potential and functional relevance.

#### RNA Expression Clustering and Pathway Analyses

The UCSC TumorMap analysis was used to visualize clusters of expression profiles across PDX histologies (Newton et al., 2017). The expression values were transformed into log_2_(TPM + 1) space. We removed genes where more than 80% of the samples had no measurable expression and we applied a variance filter to remove the 20% least varying genes. This generated a gene by sample matrix containing 28,482 genes and 244 PDX samples. The expression values and PDX annotations were uploaded to the TumorMap portal for analysis. A Bayesian hierarchical model was used to infer differences in expression across PDX histologies. We used a hierarchical modeling strategy to leverage similarities across related tissues and to improve inferences for histologies with small sample sizes (Ji and Liu, 2010). The hierarchical model was implemented using the Stan statistical programming language (Carpenter et al., 2017).

We inferred the biological function of histology-specific expression by ranking the expression differences for each histology and performing gene-set enrichment analysis (GSEA). GSEA was performed using the fgsea software (Sergushichev, 2016). Statistically significant enrichment was defined as having an adjusted p value less than 0.05 and a normalized enrichment score greater than 2.0. Statistically insignificant enrichment scores were set to zero for heatmap visualization. The normalized enrichment scores were visualized using the seaborn clustermap software for tissue database scores and R for Hallmark pathway scores.

#### Pediatric cBioPortal Data Processing

All processed data: RNA-sequencing expression values (FPKM and Z-score), RNA fusions, mutation calls in Mutation Annotation Format (MAF), segmentation, and focal copy number values were formatted using the current cBioPortal v1.2.2 file format documentation.

### DATA AND CODE AVAILABILITY

#### Raw Data Availability

Mouse and human separated DNA and RNA BAM files have been deposited into dbGAP under accession number phs001437.v1.p1.

### INTERMEDIATE PROCESSED DATA AVAILABILITY

Variant files, SNP array files, contamination assessment files: https://figshare.com/projects/Genomic_landscape_of_childhood_cancer_patient-derived_xenograft_models/38147

#### Processed Data Availability

WES mutations, mRNA expression, RNA fusions, segmentation, and gene copy number has been deposited into the publicly-available pediatric cBioportal at: https://pedcbioportal.org/study?id=pptc#summary

#### Code Created or Modified for Analysis in This Paper Have Been Deposited in GitHub

PDX mouse subtraction: https://github.com/marislab/pdx-mouse-subtraction

NGSCheckmate analysis: https://github.com/d3b-center/ngs_checkmate_wf

Correlation analyses: https://github.com/marislab/create-pptc-pdx-corplots

PDX pie chart (Figure 1): https://github.com/marislab/create-pptc-pdx-pie

Oncoprint generation (Figures 2 and 3): https://github.com/marislab/create-pptc-pdx-oncoprints

Medulloblastoma classification (Figure 2): https://github.com/PichaiRaman/MedulloClassifier

Tumor mutation burden (Figures 3 and S3): https://github.com/marislab/pptc-pdx-tmb

Gene classification (Figure 4): https://github.com/marislab/pdx-classification

Classifier analysis (Figures 4 and S5): https://github.com/marislab/pptc-pdx-classifier-analysis

RNA clustering and heatmaps (Figure 5): https://github.com/marislab/pptc-pdx-RNA-Seq-clustering

RNA fusion analysis (Figure 5): https://github.com/marislab/pptx-pdx-fusion-analysis

Ethnicity inference (Figure S2): https://github.com/marislab/pptc-pdx-ethnicity-inference

Mutational signatures (Figure S3): https://github.com/marislab/pptc-pdx-mut-sigs

Copy number, breakpoint, and SV (*ATRX* deletion) analysis (Figure S4): https://github.com/marislab/pptc-pdx-copy-number-and-SVs

## Supplementary Material

1

2

3

4

5

6

## Figures and Tables

**Table:** KEY RESOURCES TABLE

REAGENT or RESOURCE	SOURCE	IDENTIFIER
Critical Commercial Assays		
KAPA HiFi DNA Polymerase	Kapa Biosystems	KK2612
Agencourt AMPure XP beads	Beckman Coulter	A63882
SeqCap EZ HGSC VCRome Kit v 2.1	Roche	06266380001
TruSeq SBS kit v3 HS	Illumina	FC-401-3001
Oligo(dT)25 Dynabeads	Life Technologies	61002
ERCC spike-in mix #1	Ambion, Life Technologies	4456740
NEBNext RNA First Strand Synthesis Module	New England Biolabs	E7525S
NEBNext Ultra Directional RNA Second Strand Synthesis Module	New England Biolabs	E7550S
Uracil-DNA Glycosylase	New England Biolabs	M0280L
Phusion High-Fidelity PCR Master Mix	New England Biolabs	M0531L
Infinium OmniExpress-24 Kit	Illumina	WG-315-1101
GenePrint24 System for STR Typing	Promega	B1870
Investigator Quantiplex Kit	QIAGEN	387018
PrimeTime Gene Expression 2x qPCR mix	IDT	1055772
Deposited Data		
WES human and mouse BAM files	This paper	dbGAP phs001437
RNA-Seq human and mouse BAM files	This paper	dbGAP phs001437
Intermediate files	This paper	https://figshare.com/projects/Genomic_landscape_of_childhood_cancer_patient-derived_xenograft_models/38147
Processed data – somatic mutations, gene expression, RNA fusions, segmentation files, focal copy number	This paper	https://pedcbioportal.org/login.jsp#summary
Processed data – SNP array-associated analyses files, FPKM matrix, WES MAF files	This paper	Figshare
HapMap 3 draft release 2	International HapMap project	ftp://ftp.ncbi.nlm.nih.gov/hapmap/genotypes/latest_phaseIII_ncbi_b36/plink_format/
Experimental Models: Organisms/Strains		
261 pediatric PDX models	This paper	Table S1
Oligonucleotides		
Human PTGER2 qPCR FWD primer, 5′-GCTGCTTCTCATTGTCTCGG-3′	IDT	custom
Human PTGER2 qPCR REV primer, 5′-GCCAGGAGAATGAGGTGGTC-3′	IDT	custom
Human pTGER2 qPCR probe, 5′-FAM-CAGTGTCATTCTCAACCTCATCCGCA-IOWA-BLACK-3′	IDT	custom
Mouse pTGER2 qPCR FWD primer, 5′-ACATCAGCGTTATCCTCAACC-3′	IDT	custom
Mouse pTGER2 qPCR REV primer, 5′-GCTACTGCCAGACAATCCG-3′	IDT	custom
Mouse pTGER2 qPCR probe, 5′-TXRED-TCATTCGCATGCACCGTCGGA-IOWA-BLACK-3′	IDT	custom
Software and Algorithms		
FusionCatcher 0.99.7b	Nicorici et al., 2014	https://github.com/ndaniel/fusioncatcher
STAR-Fusion 1.1.0	Haas et al., 2017	https://github.com/STAR-Fusion
STAR 2.5.2b	Dobin et al., 2013	https://github.com/alexdobin/STAR
RSEM 1.2.28	Li and Dewey, 2011	https://github.com/deweylab/RSEM
TumorMap 1.0	Newton et al., 2017	https://tumormap.ucsc.edu/
Stan 2.16.0	Carpenter et al., 2017	https://github.com/stan-dev/cmdstan
Fgsea 1.5.1	Sergushichev, 2016	https://bioconductor.org/packages/release/bioc/html/fgsea.html
Pandas 0.23.0	McKinney, 2010	https://pandas.pydata.org/
R, various	R Core Team	http://www.R-project.org/
Python 3.6.5	Python Core Team	https://www.python.org/
Jupyter 1.0.0	Kluyver et al., 2016	https://jupyter.org/index.html
Seaborn 0.8.1	Seaborn Core Team	https://seaborn.pydata.org/
Maftools 2.0.15	Mayakonda et al., 2018	https://github.com/PoisonAlien/maftools
R 3.4.3	R Core Team	http://www.R-project.org/
ComplexHeatmap 2.1.0	Gu et al., 2016	https://www.bioconductor.org/packages/3.7/bioc/html/ComplexHeatmap.html
deconstructSigs 1.8.0	Rosenthal et al., 2016	https://github.com/raerose01/deconstructSigs
Nexus 8.0	Biodiscovery	https://www.biodiscovery.com/
GISTIC 2.0.23	Mermel et al., 2011	https://www.broadinstitute.org/node/358411
MutSigCV 1.3.01	Lawrence et al., 2013	http://software.broadinstitute.org/cancer/software/genepattern/modules/docs/MutSigCV
HGSC Mercury 3.2	Reid et al., 2014	https://www.hgsc.bcm.edu/software/mercury
BWA 0.7.17-r1188	Li and Durbin, 2010	http://bio-bwa.sourceforge.net/
GATK 3.8.1	McKenna et al., 2010	https://www.broadinstitute.org/gatk/
PLINK 1.9	Chang et al., 2015	https://www.cog-genomics.org/plink/1.9/
PLINK 1.07	Purcell et al., 2007	http://zzz.bwh.harvard.edu/plink/
Samtools 1.9	Li et al., 2009	http://samtools.sourceforge.net/
Sambamba 0.6.6	Tarasov et al., 2015	https://github.com/biod/sambamba
Picard 2.18.14-0	2018	https://github.com/broadinstitute/picard
Cufflinks 2.2.1	Trapnell et al., 2010	https://github.com/cole-trapnell-lab/cufflinks
RNA-SeQC 1.1.8	Deluca et al., 2012	https://github.com/broadinstitute/rnaseqc
AlignStats 0.3	BCM-HGSC	https://github.com/jfarek/alignstats
SOAPFuse 1.26	Jia et al., 2013	https://sourceforge.net/projects/soapfuse/
HTSeq 0.9.1	Anders et al., 2015	https://github.com/simon-anders/htseq
Pindel 0.2.5b5	Ye et al., 2009	https://github.com/genome/pindel
deFuse 0.7.0	McPherson et al., 2011	https://github.com/amcpherson/defuse
Bamutil 1.0.14	Jun et al., 2015	https://github.com/statgen/bamUtil
Trinity 2.5.1	Grabherr et al., 2011	https://github.com/trinityrnaseq/trinityrnaseq
Strelka 2.9.2	Kim et al., 2018b	https://github.com/Illumina/strelka
NGSCheckmate 1.0	Lee et al., 2017	https://github.com/parklab/NGSCheckMate
Other		
TARGET pediatric tumors RNA-sequencing dataset	The TARGET Consortium	https://ocg.cancer.gov/programs/target/data-matrix
GTEx normal tissues RNA-sequencing dataset	The GTEx Consortium. 2013	http://www.gtexportal.org/home/index.html
Exome Aggregation Consortium 0.3.1	Lek et al., 2016	http://exac.broadinstitute.org/
The International Genome Sample Resource and 1000 genomes project	Birney and Soranzo, 2015	https://www.internationalgenome.org/
NHBLI Exome Sequencing Project (ESP)	Exome Variant Server, NHLBI GO Exome Sequencing Project (ESP), Seattle, WA (URL: http://evs.gs.washington.edu/EVS/) [date (month, year) accessed].	http://evs.gs.washington.edu/EVS/

## Highlights

- Multiplatform analysis facilitates genomic resource of 261 pediatric cancer PDX models
- PPTC PDX models are reflective of high-risk and chemotherapy resistant disease
- Inferred TP53 pathway inactivation correlates with pediatric cancer copy number burden
- Pediatric cancer PDX models will be useful for drug development prioritization

## References

[R1] Alcoser SY, Kimmel DJ, Borgel SD, Carter JP, Dougherty KM, and Hollingshead MG (2011). Real-time PCR-based assay to quantify the relative amount of human and mouse tissue present in tumor xenografts. BMC Biotechnol. 11, 124. 22176647 10.1186/1472-6750-11-124 PMC3281124

[R2] American Childhood Cancer Organization (2014). Special Section: Cancer in Children & Adolescents In Cancer Facts and Figures 2014 (The American Cancer Society), pp. 25–42.

[R3] Anders S, Pyl PT, and Huber W (2015). HTSeq–a Python framework to work with high-throughput sequencing data. Bioinformatics 31, 166–169. 25260700 10.1093/bioinformatics/btu638 PMC4287950

[R4] Bainbridge MN, Wang M, Wu Y, Newsham I, Muzny DM, Jefferies JL, Albert TJ, Burgess DL, and Gibbs RA (2011). Targeted enrichment beyond the consensus coding DNA sequence exome reveals exons with higher variant densities. Genome Biol. 12, R68. 21787409 10.1186/gb-2011-12-7-r68 PMC3218830

[R5] Baker SC, Bauer SR, Beyer RP, Brenton JD, Bromley B, Burrill J, Causton H, Conley MP, Elespuru R, Fero M, ; External RNA Controls Consortium (2005). The External RNA Controls Consortium: a progress report. Nat. Methods 2, 731–734. 16179916 10.1038/nmeth1005-731

[R6] Behjati S, Tarpey PS, Haase K, Ye H, Young MD, Alexandrov LB, Farndon SJ, Collord G, Wedge DC, Martincorena I, (2017). Recurrent mutation of IGF signalling genes and distinct patterns of genomic rearrangement in osteosarcoma. Nat. Commun 8, 15936. 28643781 10.1038/ncomms15936 PMC5490007

[R7] Birney E, and Soranzo N (2015). Human genomics: The end of the start for population sequencing. Nature 526, 52–53. 26432243 10.1038/526052a

[R8] Boeva V, Jouannet S, Daveau R, Combaret V, Pierre-Eugène C, Cazes A, Louis-Brennetot C, Schleiermacher G, Ferrand S, Pierron G, (2013). Breakpoint features of genomic rearrangements in neuroblastoma with unbalanced translocations and chromothripsis. PLoS One 8, e72182. 23991058 10.1371/journal.pone.0072182 PMC3753337

[R9] Bosse KR, Raman P, Zhu Z, Lane M, Martinez D, Heitzeneder S, Rathi KS, Kendsersky NM, Randall M, Donovan L, (2017). Identification of GPC2 as an Oncoprotein and Candidate Immunotherapeutic Target in High-Risk Neuroblastoma. Cancer Cell 32, 295–309.e12. 28898695 10.1016/j.ccell.2017.08.003 PMC5600520

[R10] Brabetz S, Leary SES, Gröbner SN, Nakamoto MW, Şeker-Cin H, Girard EJ, Cole B, Strand AD, Bloom KL, Hovestadt V, (2018). A biobank of patient-derived pediatric brain tumor models. Nat. Med 24, 1752–1761. 30349086 10.1038/s41591-018-0207-3

[R11] Cancer Genome Atlas Research Network; Weinstein JN, Collisson EA, Mills GB, Shaw KRM, Ozenberger BA, Ellrott K, Shmulevich I, Sander C, and Stuart JM. (2013). The Cancer Genome Atlas Pan-Cancer analysis project. Nat. Genet 45, 1113–1120. 24071849 10.1038/ng.2764 PMC3919969

[R12] Carpenter B, Gelman A, Hoffman MD, Lee D, Goodrich B, Betancourt M, Brubaker M, Guo J, Li P, and Riddell A (2017). Stan: A probabilistic programming language. J. Stat. Softw 76, 1–32. 10.18637/jss.v076.i01 PMC9788645 36568334

[R13] Challis D, Yu J, Evani US, Jackson AR, Paithankar S, Coarfa C, Milosavljevic A, Gibbs RA, andYu F (2012). An integrative variant analysis suite for whole exome next-generation sequencing data. BMC Bioinformatics 13, 8. 22239737 10.1186/1471-2105-13-8 PMC3292476

[R14] Chang CC, Chow CC, Tellier LC., Vattikuti S, Purcell SM, and Lee JJ. (2015). Second-generation PLINK: rising to the challenge of larger and richer datasets. Gigascience 4, 7. 25722852 10.1186/s13742-015-0047-8 PMC4342193

[R15] Cortes-Ciriano I, Lee JK, Xi R, Jain D, Jung YL, Yang L, Gordenin D, Klimczak LJ, Zhang C-Z, Pellman DS, (2018). Comprehensive analysis of chromothripsis in 2,658 human cancers using whole-genome sequencing. bioRxiv. 10.1101/333617. PMC10300649 32404988

[R16] DeLuca DS, Levin JZ, Sivachenko A, Fennell T, Nazaire M-D, Williams C, Reich M, Winckler W, and Getz G (2012). RNA-SeQC: RNA-seq metrics for quality control and process optimization. Bioinformatics 28, 1530–1532. 22539670 10.1093/bioinformatics/bts196 PMC3356847

[R17] Dobin A, Davis CA, Schlesinger F, Drenkow J, Zaleski C, Jha S, Batut P, Chaisson M, and Gingeras TR (2013). STAR: ultrafast universal RNA-seq aligner. Bioinformatics 29, 15–21. 23104886 10.1093/bioinformatics/bts635 PMC3530905

[R18] El-Hoss J, Jing D, Evans K, Toscan C, Xie J, Lee H, Taylor RA, Lawrence MG, Risbridger GP, MacKenzie KL, (2016). A single nucleotide polymorphism genotyping platform for the authentication of patient derived xenografts. Oncotarget 7, 60475–60490. 27528024 10.18632/oncotarget.11125 PMC5312397

[R19] Eleveld TF, Oldridge DA, Bernard V, Koster J, Colmet Daage L, Diskin SJ, Schild L, Bentahar NB, Bellini A, Chicard M, (2015). Relapsed neuroblastomas show frequent RAS-MAPK pathway mutations. Nat. Genet 47, 864–871. 26121087 10.1038/ng.3333 PMC4775079

[R20] Gelman A (2006). Multilevel (Hierarchical) Modeling: What It Can and Cannot Do. Technometrics 48, 432–35.

[R21] Grabherr MG, Haas BJ, Yassour M, Levin JZ, Thompson DA, Amit I, Adiconis X, Fan L, Raychowdhury R, Zeng Q, (2011). Full-length transcriptome assembly from RNA-Seq data without a reference genome. Nat. Biotechnol 29, 644–652. 21572440 10.1038/nbt.1883 PMC3571712

[R22] Gröbner SN, Worst BC, Weischenfeldt J, Buchhalter I, Kleinheinz K, Rudneva VA, Johann PD, Balasubramanian GP, Segura-Wang M, Brabetz S, (2018). The landscape of genomic alterations across childhood cancers. Nature 555, 321–327. 29489754 10.1038/nature25480

[R23] GTEx Consortium (2013). The Genotype-Tissue Expression (GTEx) project. Nat. Genet 45, 580–585. 23715323 10.1038/ng.2653 PMC4010069

[R24] Gu Z, Eils R, and Schlesner M (2016). Complex heatmaps reveal patterns and correlations in multidimensional genomic data. Bioinformatics 32, 2847–2849. 27207943 10.1093/bioinformatics/btw313

[R25] Haas BJ, Dobin A, Stransky N, Li B, Yang X, Tickle T, Bankapur A, Ganote C, Doak TG, Pochet N, (2017). STAR-Fusion: Fast and Accurate Fusion Transcript Detection from RNA-Seq. bioRxiv. 10.1101/120295.

[R26] Houghton PJ, Adamson PC, Blaney S, Fine HA, Gorlick R, Haber M, Helman L, Hirschfeld S, Hollingshead MG, Israel MA, (2002). Testing of new agents in childhood cancer preclinical models: meeting summary. Clin. Cancer Res 8, 3646–3657. 12473573

[R27] Houghton PJ, Morton CL, Tucker C, Payne D, Favours E, Cole C, Gorlick R, Kolb EA, Zhang W, Lock R, (2007). The pediatric preclinical testing program: description of models and early testing results. Pediatr. Blood Cancer 49, 928–940. 17066459 10.1002/pbc.21078

[R28] Ijaz H, Koptra M, Gaonkar KS, Rokita J, Baubet VP, Yauhid L, Zhu Y, Brown M, Lopez G, Zhang B, (2019). Pediatric High Grade Glioma Resources From The Children’s Brain Tumor Tissue Consortium (CBTTC) And Pediatric Brain Tumor Atlas (PBTA). bioRxiv. 10.1101/656587. PMC6954395 31613963

[R29] Ji H, and Liu XS. (2010). Analyzing ’omics data using hierarchical models. Nat. Biotechnol 28, 337–340. 20379180 10.1038/nbt.1619 PMC2904972

[R30] Jia W, Qiu K, He M, Song P, Zhou Q, Zhou F, Yu Y, Zhu D, Nickerson ML, Wan S, (2013). SOAPfuse: an algorithm for identifying fusion transcripts from paired-end RNA-Seq data. Genome Biol. 14, R12. 23409703 10.1186/gb-2013-14-2-r12 PMC4054009

[R31] Jun G, Wing MK, Abecasis GR, and Kang HM (2015). An efficient and scalable analysis framework for variant extraction and refinement from population-scale DNA sequence data. Genome Res. 25, 918–925. 25883319 10.1101/gr.176552.114 PMC4448687

[R32] Kim P, Park A, Han G, Sun H, Jia P, and Zhao Z (2018). TissGDB: tissue-specific gene database in cancer. Nucleic Acids Res. 46 (D1), D1031–D1038. 29036590 10.1093/nar/gkx850 PMC5753286

[R33] Kim S, Scheffler K, Halpern AL, Bekritsky MA, Noh E, Källberg M, Chen X, Kim Y, Beyter D, Krusche P, (2018). Strelka2: fast and accurate calling of germline and somatic variants. Nat. Methods 15, 591–594. 30013048 10.1038/s41592-018-0051-x

[R34] Kluyver T, Ragan-Kelley B, Pérez F, Granger BE, Bussonnier M, Frederic J, Kelley K, Hamrick JB, Grout J, Corlay S, (2016). Jupyter Notebooks-a publishing format for reproducible computational workflows In Positioning and Power in Acadmic Publishing: Players, Agents and Agendas, Loizides F and Scmidt B, eds. (IOS Press), pp. 87–90.

[R35] Knijnenburg TA, Wang L, Zimmermann MT, Chambwe N, Gao GF, Cherniack AD, Fan H, Shen H, Way GP, Greene CS, ; Cancer Genome Atlas Research Network (2018). Genomic and Molecular Landscape of DNA Damage Repair Deficiency across The Cancer Genome Atlas. Cell Rep. 23, 239–254.e6. 29617664 10.1016/j.celrep.2018.03.076 PMC5961503

[R36] Lawrence MS, Stojanov P, Polak P, Kryukov GV, Cibulskis K, Sivachenko A, Carter SL, Stewart C, Mermel CH, Roberts SA, (2013). Mutational heterogeneity in cancer and the search for new cancer-associated genes. Nature 499, 214–218. 23770567 10.1038/nature12213 PMC3919509

[R37] Lecona E, and Fernandez-Capetillo O (2018). Targeting ATR in cancer. Nat. Rev. Cancer 18, 586–595. 29899559 10.1038/s41568-018-0034-3

[R38] Lee S, Lee S, Ouellette S, Park W-Y, Lee EA, and Park PJ (2017). NGSCheckMate: software for validating sample identity in next-generation sequencing studies within and across datatypes. Nucleic Acids Res. 45, e103. 28369524 10.1093/nar/gkx193 PMC5499645

[R39] Lek M, Karczewski KJ, Minikel EV, Samocha KE, Banks E, Fennell T, O’Donnell-Luria AH, Ware JS, Hill AJ, Cummings BB, ; Exome Aggregation Consortium (2016). Analysis of protein-coding genetic variation in 60,706 humans. Nature 536, 285–291. 27535533 10.1038/nature19057 PMC5018207

[R40] Li B, and Dewey CN (2011). RSEM: accurate transcript quantification from RNA-Seq data with or without a reference genome. BMC Bioinformatics 12, 323. 21816040 10.1186/1471-2105-12-323 PMC3163565

[R41] Li H, and Durbin R (2010). Fast and accurate long-read alignment with Burrows-Wheeler transform. Bioinformatics 26, 589–595. 20080505 10.1093/bioinformatics/btp698 PMC2828108

[R42] Li H, Handsaker B, Wysoker A, Fennell T, Ruan J, Homer N, Marth G, Abecasis G, and Durbin R; 1000 Genome Project Data Processing Subgroup (2009). The Sequence Alignment/Map format and SAMtools. Bioinformatics 25, 2078–2079. 19505943 10.1093/bioinformatics/btp352 PMC2723002

[R43] Liem NLM, Papa RA, Milross CG, Schmid MA, Tajbakhsh M, Choi S, Ramirez CD, Rice AM, Haber M, Norris MD, (2004). Characterization of childhood acute lymphoblastic leukemia xenograft models for the pre-clinical evaluation of new therapies. Blood 103, 3905–3914. 14764536 10.1182/blood-2003-08-2911

[R44] Liu X, Yu X, Zack DJ, Zhu H, and Qian J (2008). TiGER: a database for tissue-specific gene expression and regulation. BMC Bioinformatics 9, 271. 18541026 10.1186/1471-2105-9-271 PMC2438328

[R45] Liu Y, Easton J, Shao Y, Maciaszek J, Wang Z, Wilkinson MR, McCastlain K, Edmonson M, Pounds SB, Shi L, (2017). The genomic landscape of pediatric and young adult T-lineage acute lymphoblastic leukemia. Nat. Genet 49, 1211–1218. 28671688 10.1038/ng.3909 PMC5535770

[R46] Lock RB, Liem N, Farnsworth ML, Milross CG, Xue C, Tajbakhsh M, Haber M, Norris MD, Marshall GM, and Rice AM (2002). The nonobese diabetic/severe combined immunodeficient (NOD/SCID) mouse model of childhood acute lymphoblastic leukemia reveals intrinsic differences in biologic characteristics at diagnosis and relapse. Blood 99, 4100–4108. 12010813 10.1182/blood.v99.11.4100

[R47] Lorenz S, Barøy T, Sun J, Nome T, Vodák D, Bryne J-C, Håkelien A-M, Fernandez-Cuesta L, Möhlendick B, Rieder H, (2016). Unscrambling the genomic chaos of osteosarcoma reveals extensive transcript fusion, recurrent rearrangements and frequent novel TP53 aberrations. Oncotarget 7, 5273–5288. 26672768 10.18632/oncotarget.6567 PMC4868685

[R48] Ma X, Edmonson M, Yergeau D, Muzny DM, Hampton OA, Rusch M, Song G, Easton J, Harvey RC, Wheeler DA, (2015). Rise and fall of subclones from diagnosis to relapse in pediatric B-acute lymphoblastic leukaemia. Nat. Commun 6, 6604. 25790293 10.1038/ncomms7604 PMC4377644

[R49] Ma X, Liu Y, Liu Y, Alexandrov LB, Edmonson MN, Gawad C, Zhou X, Li Y, Rusch MC, Easton J, (2018). Pan-cancer genome and transcriptome analyses of1,699 paediatric leukaemias and solid tumours. Nature 555, 371–376. 29489755 10.1038/nature25795 PMC5854542

[R50] Mackay A, Burford A, Carvalho D, Izquierdo E, Fazal-Salom J, Taylor KR, Bjerke L, Clarke M, Vinci M, Nandhabalan M, (2017). Integrated Molecular Meta-Analysis of 1,000 Pediatric High-Grade and Diffuse Intrinsic Pontine Glioma. Cancer Cell 32, 520–537.e5. 28966033 10.1016/j.ccell.2017.08.017 PMC5637314

[R51] Mayakonda A, Lin D-C, Assenov Y, Plass C, and Koeffler HP (2018). Maftools: efficient and comprehensive analysis of somatic variants in cancer. Genome Res. 28, 1747–1756. 30341162 10.1101/gr.239244.118 PMC6211645

[R52] McKenna A, Hanna M, Banks E, Sivachenko A, Cibulskis K, Kernytsky A, Garimella K, Altshuler D, Gabriel S, Daly M, (2010). The Genome Analysis Toolkit: a MapReduce framework for analyzing next-generation DNA sequencing data. Genome Res. 20, 1297–1303. 20644199 10.1101/gr.107524.110 PMC2928508

[R53] McKinney W (2010). Data structures for statistical computing in python. In Proceedings of the 9th Python in Science Conference (SCIPY), pp. 51–56.

[R54] McPherson A, Hormozdiari F, Zayed A, Giuliany R, Ha G, Sun MGF, Griffith M, Heravi Moussavi A, Senz J, Melnyk N, (2011). deFuse: An Algorithm for Gene Fusion Discovery in Tumor RNA-Seq Data. PLoS Comput. Biol 7, e1001138. 21625565 10.1371/journal.pcbi.1001138 PMC3098195

[R55] Mermel CH, Schumacher SE, Hill B, Meyerson ML, Beroukhim R, and Getz G (2011). GISTIC2.0 facilitates sensitive and confident localization of the targets of focal somatic copy-number alteration in human cancers. Genome Biol. 12, R41. 21527027 10.1186/gb-2011-12-4-r41 PMC3218867

[R56] Molenaar JJ, Koster J, Zwijnenburg DA, van Sluis P, Valentijn LJ, van der Ploeg I, Hamdi M, van Nes J, Westerman BA, van Arkel J, (2012). Sequencing of neuroblastoma identifies chromothripsis and defects in neuritogenesis genes. Nature 483, 589–593. 22367537 10.1038/nature10910

[R57] Newton Y, Novak AM, Swatloski T, McColl DC, Chopra S, Graim K, Weinstein AS, Baertsch R, Salama SR, Ellrott K, (2017). TumorMap: Exploring the Molecular Similarities of Cancer Samples in an Interactive Portal. Cancer Res. 77, e111–e114. 29092953 10.1158/0008-5472.CAN-17-0580 PMC5751940

[R58] Nicorici D, Satalan M, Edgren H, and Kangaspeska S (2014). FusionCatcher-a tool for finding somatic fusion genes in paired-end RNA-sequencing data. bioRxiv. 10.1101/011650.

[R59] Padovan-Merhar OM, Raman P, Ostrovnaya I, Kalletla K, Rubnitz KR, Sanford EM, Ali SM, Miller VA, Mossé YP, Granger MP, (2016). Enrichment of Targetable Mutations in the Relapsed Neuroblastoma Genome. PLoS Genet. 12, e1006501. 27997549 10.1371/journal.pgen.1006501 PMC5172533

[R60] Pan Z, He H, Tang L, Bu Q, Cheng H, Wang A, Lyu J, and You H (2017). Loss of heterozygosity on chromosome 16q increases relapse risk in Wilms’ tumor: a meta-analysis. Oncotarget 8, 66467–66475. 29029528 10.18632/oncotarget.20191 PMC5630428

[R61] Peters TL, Kumar V, Polikepahad S, Lin FY, Sarabia SF, Liang Y, Wang W-L, Lazar AJ, Doddapaneni H, Chao H, (2015). BCOR-CCNB3 fusions are frequent in undifferentiated sarcomas of male children. Mod. Pathol 28, 575–586. 25360585 10.1038/modpathol.2014.139 PMC4385430

[R62] Pugh TJ, Morozova O, Attiyeh EF, Asgharzadeh S, Wei JS, Auclair D, Carter SL, Cibulskis K, Hanna M, Kiezun A, (2013). The genetic landscape of high-risk neuroblastoma. Nat. Genet 45, 279–284. 23334666 10.1038/ng.2529 PMC3682833

[R63] Purcell S, Neale B, Todd-Brown K, Thomas L, Ferreira MAR, Bender D, Maller J, Sklar P, de Bakker PIW, Daly MJ, (2007). PLINK: a tool set for whole-genome association and population-based linkage analyses. Am. J. Hum. Genet 81, 559–575. 17701901 10.1086/519795 PMC1950838

[R64] Rausch T, Jones DTW, Zapatka M, Stütz AM, Zichner T, Weischenfeldt J, Jäger N, Remke M, Shih D, Northcott PA, (2012). Genome sequencing of pediatric medulloblastoma links catastrophic DNA rearrangements with TP53 mutations. Cell 148, 59–71. 22265402 10.1016/j.cell.2011.12.013 PMC3332216

[R65] Reid JG, Carroll A, Veeraraghavan N, Dahdouli M, Sundquist A, English A, Bainbridge M, White S, Salerno W, Buhay C, (2014). Launching genomics into the cloud: deployment of Mercury, a next generation sequence analysis pipeline. BMC Bioinformatics 15, 30. 24475911 10.1186/1471-2105-15-30 PMC3922167

[R66] Ribi S, Baumhoer D, Lee K, Edison, Teo AS, Madan B, Zhang K, Kohlmann WK, Yao F, Lee WH, (2015). TP53 intron 1 hotspot rearrangements are specific to sporadic osteosarcoma and can cause Li-Fraumeni syndrome. Oncotarget 6, 7727–7740. 25762628 10.18632/oncotarget.3115 PMC4480712

[R67] Rosenthal R, McGranahan N, Herrero J, Taylor BS, and Swanton C (2016). DeconstructSigs: delineating mutational processes in single tumors distinguishes DNA repair deficiencies and patterns of carcinoma evolution. Genome Biol. 17, 31. 26899170 10.1186/s13059-016-0893-4 PMC4762164

[R68] Sano R, Krytska K, Larmour CE, Raman P, Martinez D, Ligon GF, Lillquist JS, Cucchi U, Orsini P, Rizzi S, (2019). An antibody-drug conjugate directed to the ALK receptor demonstrates efficacy in preclinical models of neuroblastoma. Sci. Transl. Med 11, eaau9732. 30867324 10.1126/scitranslmed.aau9732 PMC10023134

[R69] Schleiermacher G, Javanmardi N, Bernard V, Leroy Q, Cappo J, Rio Frio T, Pierron G, Lapouble E, Combaret V, Speleman F, (2014). Emergence of new ALK mutations at relapse of neuroblastoma. J. Clin. Oncol 32, 2727–2734. 25071110 10.1200/JCO.2013.54.0674

[R70] Schramm A, Köster J, Assenov Y, Althoff K, Peifer M, Mahlow E, Odersky A, Beisser D, Ernst C, Henssen AG, (2015). Mutational dynamics between primary and relapse neuroblastomas. Nat. Genet 47, 872–877. 26121086 10.1038/ng.3349

[R71] Scott RH, Murray A, Baskcomb L, Turnbull C, Loveday C, Al-Saadi R, Williams R, Breatnach F, Gerrard M, Hale J, (2012). Stratification of Wilms tumor by genetic and epigenetic analysis. Oncotarget 3, 327–335. 22470196 10.18632/oncotarget.468 PMC3359888

[R72] Segers H, van den Heuvel-Eibrink MM, Williams RD, van Tinteren H, Vujanic G, Pieters R, Pritchard-Jones K, and Bown N; Children’s Cancer and Leukaemia Group and the UK Cancer Cytogenetics Group(2013). Gain of 1 q is a marker of poor prognosis in Wilms’ tumors. Genes Chromosomes Cancer 52, 1065–1074. 24038759 10.1002/gcc.22101

[R73] Sergushichev AA (2016). An algorithm for fast preranked gene set enrichment analysis using cumulative statistic calculation. bioRxiv. 10.1101/060012.

[R74] Shen Y, Wan Z, Coarfa C, Drabek R, Chen L, Ostrowski EA, Liu Y, Weinstock GM, Wheeler DA, Gibbs RA, and Yu F (2010).ASNP discovery method to assess variant allele probability from next-generation resequencing data. Genome Res. 20, 273–280. 20019143 10.1101/gr.096388.109 PMC2813483

[R75] Shern JF, Chen L, Chmielecki J, Wei JS, Patidar R, Rosenberg M, Ambrogio L, Auclair D, Wang J, Song YK, (2014). Comprehensive genomic analysis of rhabdomyosarcoma reveals a landscape of alterations affecting a common genetic axis in fusion-positive and fusion-negative tumors. Cancer Discov. 4, 216–231. 24436047 10.1158/2159-8290.CD-13-0639 PMC4462130

[R76] Spreafico F, Gamba B, Mariani L, Collini P, D’Angelo P, Pession A, Di Cataldo A, Indolfi P, Nantron M, Terenziani M, ; AIEOP Wilms Tumor Working Group (2013). Loss of heterozygosity analysis at different chromosome regions in Wilms tumor confirms 1p allelic loss as a marker of worse prognosis: a study from the Italian Association of Pediatric Hematology and Oncology. J. Urol 189, 260–266. 23174227 10.1016/j.juro.2012.09.009

[R77] Stewart E, Federico SM, Chen X, Shelat AA, Bradley C, Gordon A, Karlstrom A, Twarog NR, Clay MR, Bahrami A, (2017). Orthotopic patient-derived xenografts of paediatric solid tumours. Nature 549, 96–100. 28854174 10.1038/nature23647 PMC5659286

[R78] Tarasov A, Vilella AJ, Cuppen E, Nijman IJ, and Prins P (2015). Sambamba: fast processing of NGS alignment formats. Bioinformatics 31, 2032–2034. 25697820 10.1093/bioinformatics/btv098 PMC4765878

[R79] Tirode F, Surdez D, Ma X, Parker M, Le Deley MC, Bahrami A, Zhang Z, Lapouble E, Grossetête-Lalami S, Rusch M, ; St. Jude Children’s Research Hospital–Washington University Pediatric Cancer Genome Project and the International Cancer Genome Consortium (2014). Genomic landscape of Ewing sarcoma defines an aggressive subtype with co-association of STAG2 and TP53 mutations. Cancer Discov. 4, 1342–1353. 25223734 10.1158/2159-8290.CD-14-0622 PMC4264969

[R80] Townsend EC, Murakami MA, Christodoulou A, Christie AL, Köster J, DeSouza TA, Morgan EA, Kallgren SP, Liu H, Wu S-C, (2016). The Public Repository of Xenografts Enables Discovery and Randomized Phase II-like Trials in Mice. Cancer Cell 29, 574–586. 27070704 10.1016/j.ccell.2016.03.008 PMC5177991

[R81] Trapnell C, Williams BA, Pertea G, Mortazavi A, Kwan G, van Baren MJ, Salzberg SL, Wold BJ, and Pachter L (2010). Transcript assembly and quantification by RNA-Seq reveals unannotated transcripts and isoform switching during cell differentiation. Nat. Biotechnol 28, 511–515. 20436464 10.1038/nbt.1621 PMC3146043

[R82] Wang L, Ni X, Covington KR, Yang BY, Shiu J, Zhang X, Xi L, Meng Q, Langridge T, Drummond J, (2015). Genomic profiling of Sézary syndrome identifies alterations of key T cell signaling and differentiation genes. Nat. Genet 47, 1426–1434. 26551670 10.1038/ng.3444 PMC4829974

[R83] Way GP, Allaway RJ, Bouley SJ, Fadul CE, Sanchez Y, and Greene AS (2017). A machine learning classifier trained on cancer transcriptomes detects NF1 inactivation signal in glioblastoma. BMC Genomics 18, 127. 28166733 10.1186/s12864-017-3519-7 PMC5292791

[R84] Way GP, Sanchez-Vega F, La K, Armenia J, Chatila WK, Luna A, Sander C, Cherniack AD, Mina M, Ciriello G, ; Cancer Genome Atlas Research Network (2018). Machine Learning Detects Pan-cancer Ras Pathway Activation in The Cancer Genome Atlas. Cell Rep. 23, 172–180.e3. 29617658 10.1016/j.celrep.2018.03.046 PMC5918694

[R85] Whiteford CC, Bilke S, Greer BT, Chen Q, Braunschweig TA, Cenacchi N, Wei JS, Smith MA, Houghton P, Morton C, (2007). Credentialing preclinical pediatric xenograft models using gene expression and tissue microarray analysis. Cancer Res. 67, 32–40. 17210681 10.1158/0008-5472.CAN-06-0610

[R86] Ye K, Schulz MH, Long Q, Apweiler R, and Ning Z (2009). Pindel: a pattern growth approach to detect break points of large deletions and medium sized insertions from paired-end short reads. Bioinformatics 25, 2865–2871. 19561018 10.1093/bioinformatics/btp394 PMC2781750

[R87] Yu L, Baxter PA, Voicu H, Gurusiddappa S, Zhao Y, Adesina A, Man T-K, Shu Q, Zhang Y-J, Zhao X-M, (2010). A clinically relevant orthotopic xenograft model of ependymoma that maintains the genomic signature of the primary tumor and preserves cancer stem cells in vivo. Neuro-oncol. 12, 580–594. 20511191 10.1093/neuonc/nop056 PMC2940646

[R88] Zhang J, Ding L, Holmfeldt L, Wu G, Heatley SL, Payne-Turner D, Easton J, Chen X, Wang J, Rusch M, (2012). The genetic basis of early T-cell precursor acute lymphoblastic leukaemia. Nature 481, 157–163. 22237106 10.1038/nature10725 PMC3267575

