# Supplementary material for: Genomic Profiling of Childhood Tumor Patient-Derived Xenograft Models to Enable Rational Clinical Trial Design
Source: Cell Rep. Author manuscript; Available in PMC 2019 Nov 27. (PMC6880934; doi:10.1016/j.celrep.2019.09.071)
Supplement: 1 [file NIHMS1542708-supplement-1.pdf]

Cell Reports, Volume *29*

Supplemental Information

Genomic Profiling of Childhood

Tumor Patient-Derived Xenograft Models

to Enable Rational Clinical Trial Design

Jo Lynne Rokita, Komal S. Rathi, Maria F. Cardenas, Kristen A. Upton, Joy Jayaseelan, Katherine L. Cross, Jacob Pfeil, Laura E. Egolf, Gregory P. Way, Alvin Farrel, Nathan M. Kendsersky, Khushbu Patel, Krutika S. Gaonkar, Apexa Modi, Esther R. Berko, Gonzalo Lopez, Zalman Vaksman, Chelsea Mayoh, Jonas Nance, Kristyn McCoy, Michelle Haber, Kathryn Evans, Hannah McCalmont, Katerina Bendak, Julia W. Böhm, Glenn M. Marshall, Vanessa Tyrrell, Karthik Kalletla, Frank K. Braun, Lin Qi, Yunchen Du, Huiyuan Zhang, Holly B. Lindsay, Sibo Zhao, Jack Shu, Patricia Baxter, Christopher Morton, Dias Kurmashev, Siyuan Zheng, Yidong Chen, Jay Bowen, Anthony C. Bryan, Kristen M. Leraas, Sara E. Coppens, HarshaVardhan Doddapaneni, Zeineen Momin, Wendong Zhang, Gregory I. Sacks, Lori S. Hart, Kateryna Krytska, Yael P. Mosse, Gregory J. Gatto, Yolanda Sanchez, Casey S. Greene, Sharon J. Diskin, Olena Morozova Vaske, David Haussler, Julie M. Gastier-Foster, E. Anders Kolb, Richard Gorlick, Xiao-Nan Li, C. Patrick Reynolds, Raushan T. Kurmasheva, Peter J. Houghton, Malcolm A. Smith, Richard B. Lock, Pichai Raman, David A. Wheeler, and John M. Maris

Figure S1, related to Figures 1-5

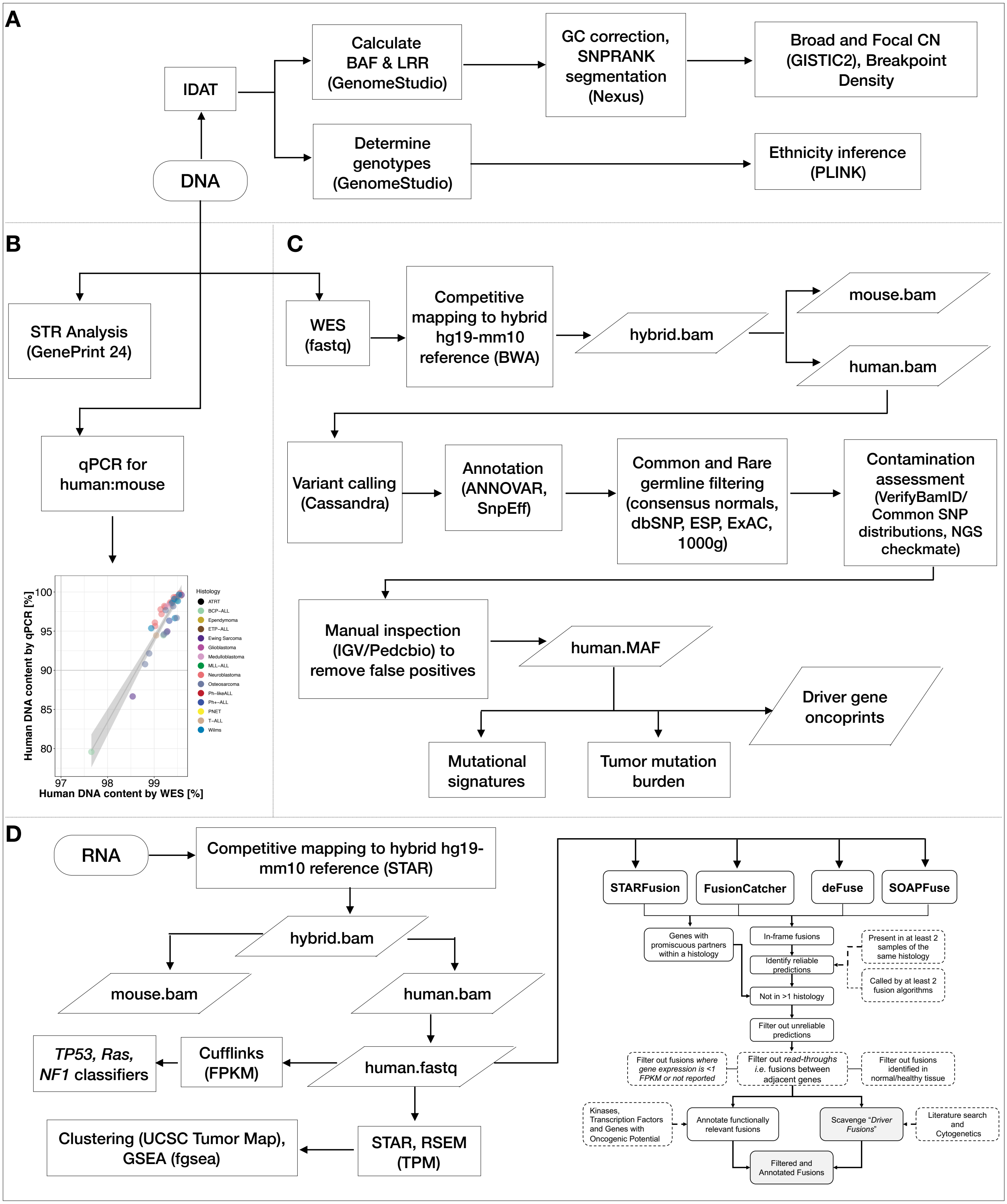

Figure S1. Analysis pipeline for somatic mutations, gene expression, RNA fusions, and copy number profiling in pediatric PDX tumors, Related to Figures 1-5. Figure S1 displays an overview of analysis methods utilized. Genomic DNA from PDX tumors was used for SNP array copy number analysis (A, N = 252), short-tandem repeat identity testing (B, N = 261), quantitative PCR to assess human:mouse DNA content (B, N = 35 samples with N = 3 technical replicates), and whole exome sequencing (C, N = 240). Total RNA from PDX tumors was used for whole transcriptome sequencing (D, N = 244). See Table S1 for Ns per assay per histology and Table S2 for STR profiles. Unless otherwise noted, Ns denote biological replicates.

Figure S2, related to Figure 1

A

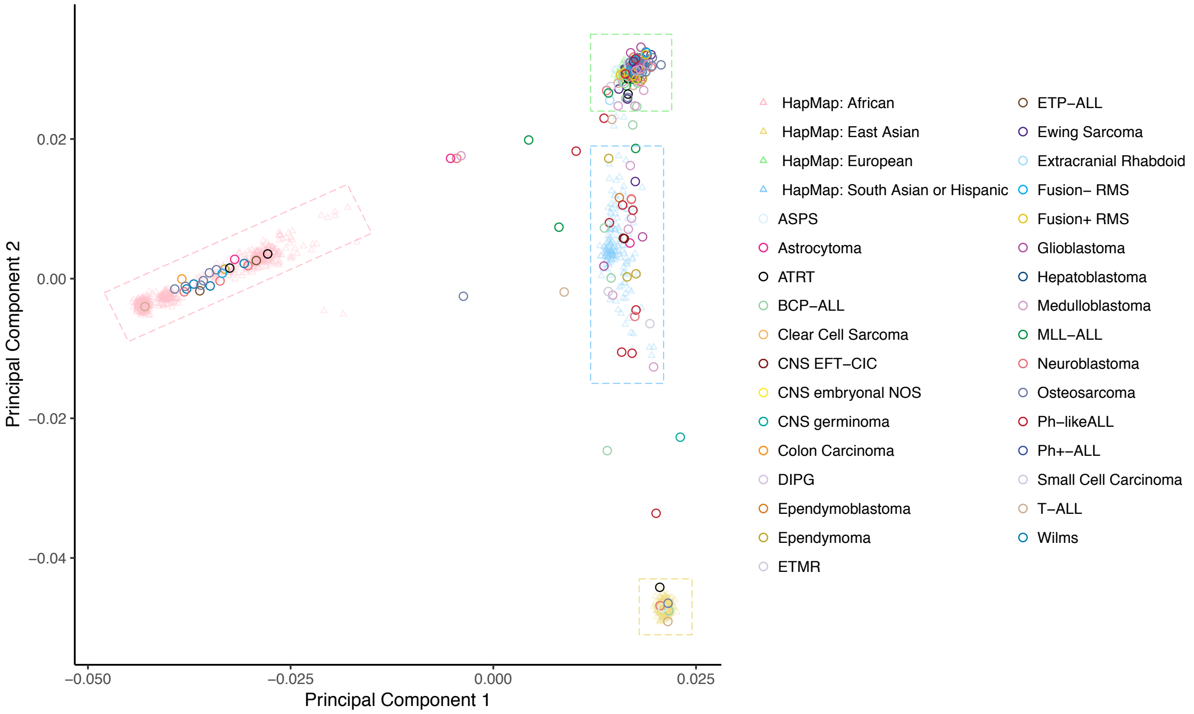

B

	Number of Models	% of Total
**European**	181	71.8%
**African**	22	8.7%
**East Asian**	6	2.4%
**South Asian or Hispanic**	29	11.5%
**Mixed or Unknown**	14	5.6%
**Total**	**252**	100%

C

		Reported Ethnicity						
		African American	European	Hispanic or Latino	Mixed	Non-Hispanic	Other	Unknown
Inferred Ethnicity	African	5	1	0	0	2	0	14
	EastAsian	0	0	0	1	0	0	5
	European	3	25	3	0	10	1	139
	Mixed or Unknown	0	0	1	0	0	1	12
	SouthAsianOrHispanic	0	0	12	0	2	0	15

Figure S2. Ethnicity prediction, Related to Figure 1. Principal components analysis grouping of European, African, East Asian, and South Asian/Hispanic HapMap reference populations used to predict PDX ethnicities (A). The first two principal components calculated from SNP array genotypes for PDX models (circles, N = 252) are plotted alongside HapMap reference samples (triangles, N = 1,184). Dashed boxes represent the cutoffs used to classify PDXs into four broad population groups: European (including HapMap CEU and TSI population samples), African (ASW, LWK, MKK, and YRI), East Asian (CHB, CHD, and JPT), and South Asian or Hispanic (GIH and MXL). Tabulated counts and frequencies of ethnicities in PDX cohort (B) and a comparison table of reported versus inferred ethnicities in the PDX cohort (C). Ns represent biological replicates.

Figure S3, related to Figure 2

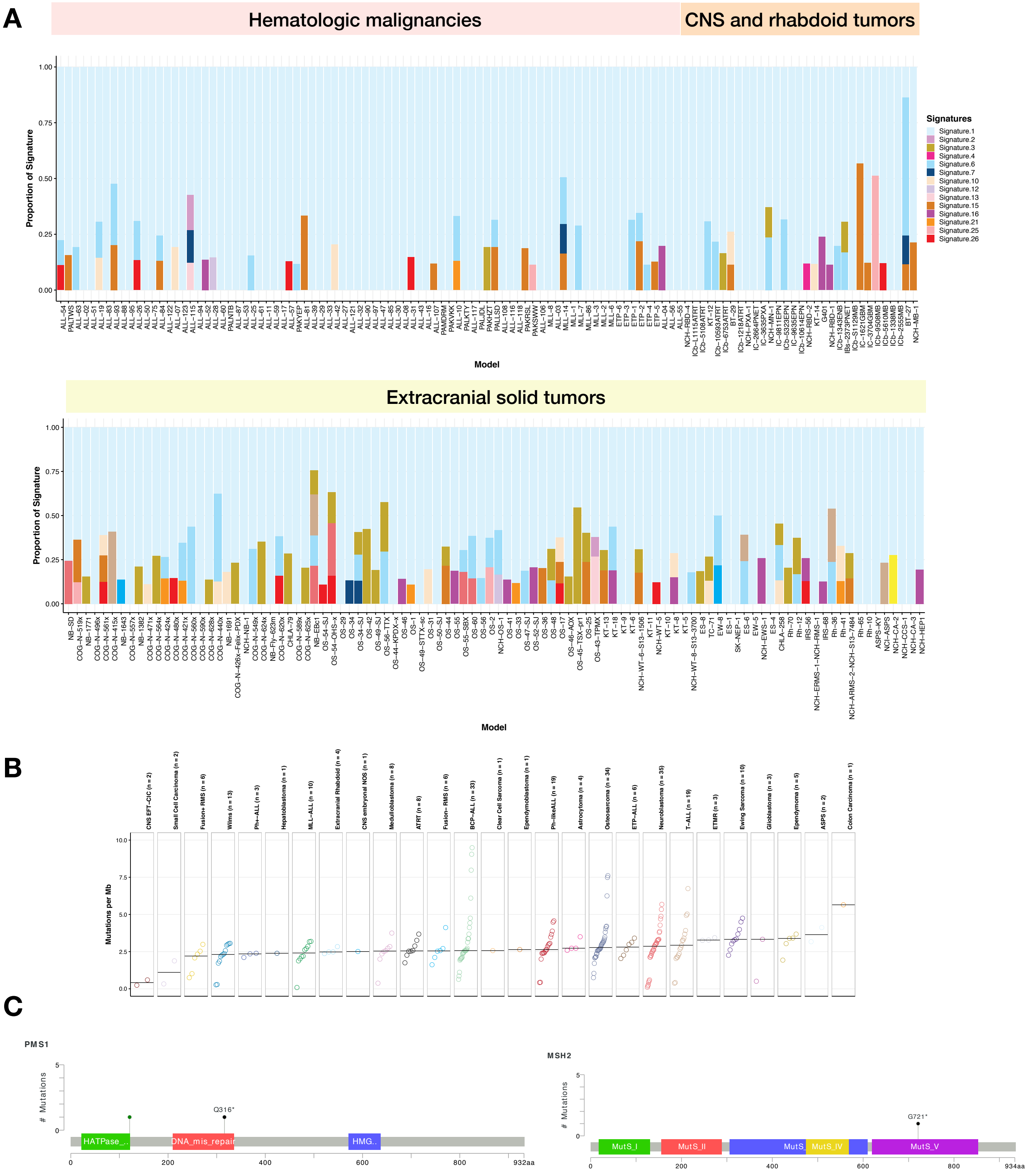

Figure S3. Mutational signatures and tumor mutational burden, Related to Figure 2. Mutational signatures per model displayed as proportion of signatures in a stacked barplot (A) Models with ≥ 50 mutations are depicted ($N_{leukemia} = 82$, $N_{brain} = 32$, $N_{solid} = 98$). Tumor mutation burden by histology across N = 240 models on which WES was performed (B, STAR methods). Histologies are plotted in rank order by median (y-intercept) and Ns per histology are listed. Lollipop plots for oncogenic mutations in DNA repair genes, *PMS1* and *MSH2* for hypermutated model, IC-1621GBM (C). Ns represent biological replicates.

Figure S4, related to Figure 2

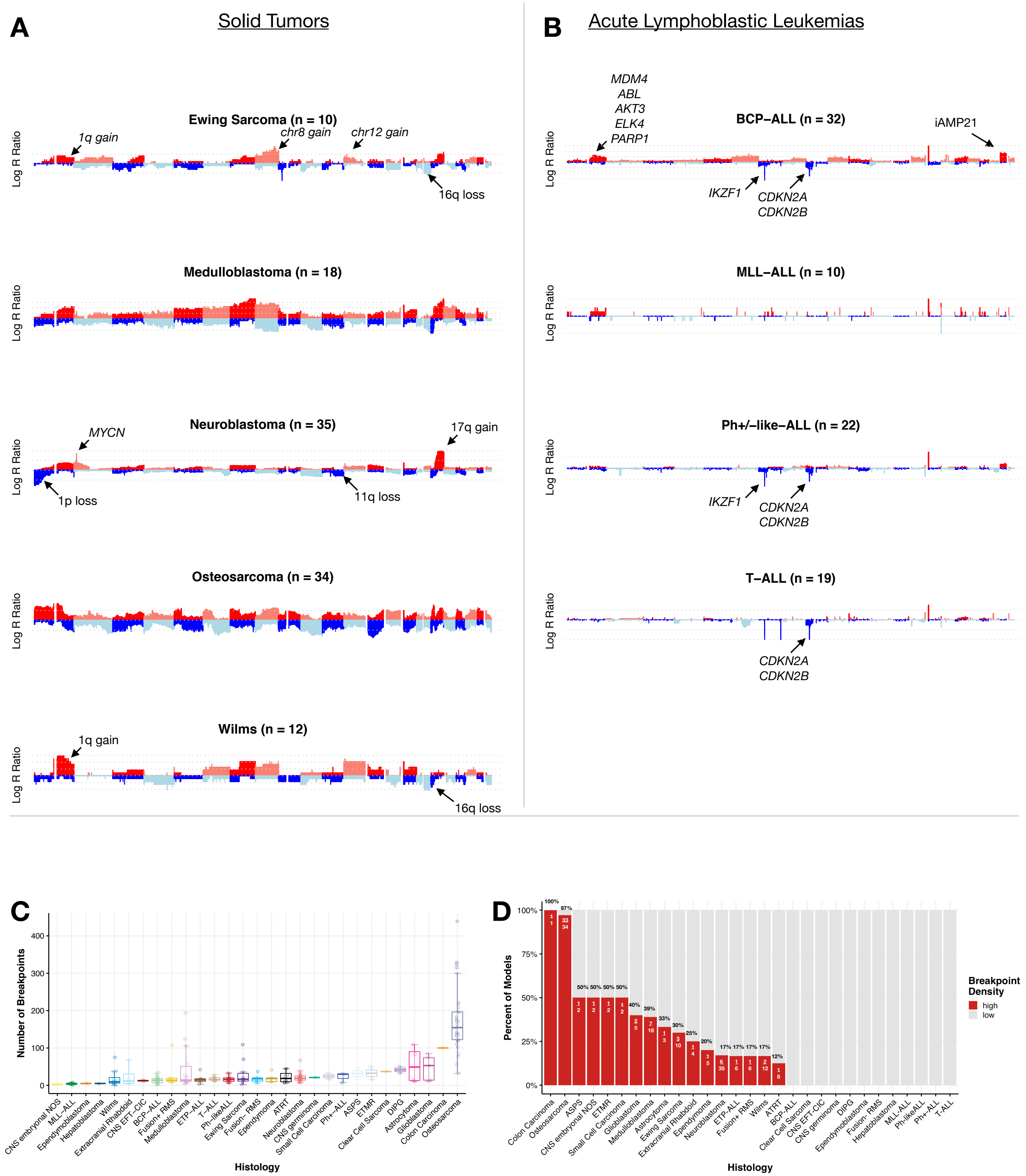

Figure S4. Copy number and breakpoint density. Related to Figure 2. Plotted are genome-wide copy-number profiles for histologies with N ≧ 10 models (Panel A, solid tumors: Ewing sarcoma, N = 10; Medulloblastoma, N = 18; Neuroblastoma, N = 35; Osteosarcoma, N = 34; Wilms, N = 12 and Panel B, leukemias: BCP-ALL, N = 32; MLL-ALL, N = 10; Ph+ or Ph-like ALL, N = 22; T-ALL, N = 19). Canonical broad and focal lesions are annotated by histology. Breakpoints per histology are plotted in C (boxplots are graphed as medians with box edges as first and third quartiles; detailed Ns in Table S4) and breakpoint density across histologies is plotted in D (displayed as % of models per histology with N/total; details in Table S4). Ns represent biological replicates.

Figure S5, related to Figures 4 and 5

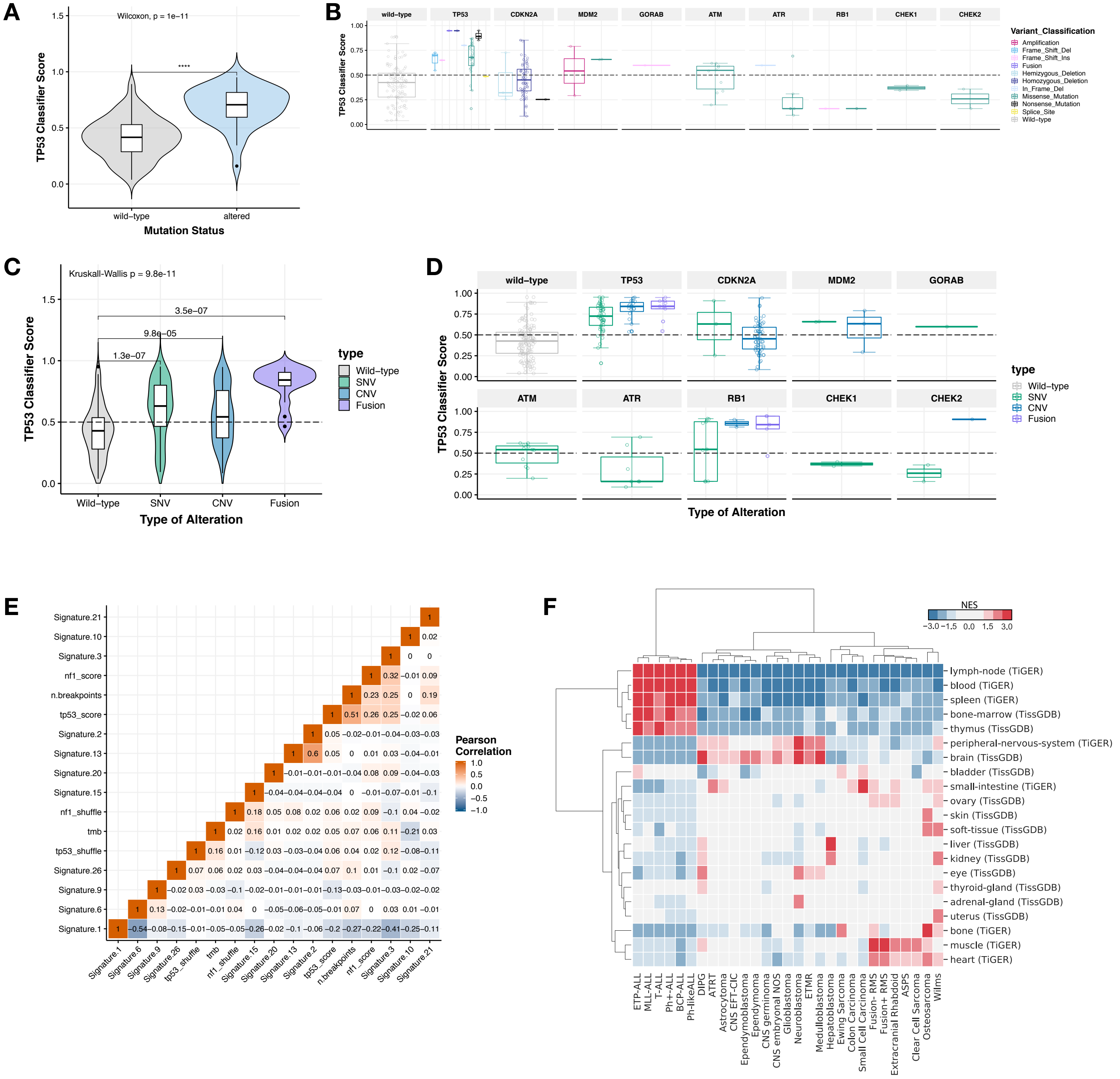

Figure S5. Classifier scores and mutational signature correlations, Related to Figure 4. With osteosarcoma models removed from analysis, *TP53* classifier scores were still significantly higher (N_{WT} = 180, N_{ALT} = 34, Wilcoxon p = 1e-11) in models with a *TP53* alteration (A), but alterations in other pathway genes don't consistently phenocopy *TP53* inactivation (B). Models containing fusions had highest classifier scores, followed by models with SNVs and CNVs, respectively (C, Kruskal-Wallis p = 9.8e-11, N_{WT} = 120, $N_{FUSIONS}$ = 14, N_{SNV} = 81, N_{CNV} = 85). *Post hoc* Wilcoxon p-values and group comparisons are displayed. Panel D breaks down the data in C by gene. Validation of mutational signatures via Pearson correlation matrix: Signatures 2 and 13 correlate strongly (R = 0.6, p= 6.5e-25, N = 260), Signature 1 is inversely correlated with impaired DNA repair mutational signatures, 3 (R = -0.41, p = 3.29e-11 , N = 260) and 6 (R = -0.54, p = 8.12e-20, N = 260) (E). Hierarchical clustering depicts tissue-specific enrichment within each histology (F, N = 244, NES = normalized enrichment score). All Ns denote biological replicates.
